# Glioma IL13Rα2 Is Associated with Mesenchymal Signature Gene Expression and Poor Patient Prognosis

**DOI:** 10.1371/journal.pone.0077769

**Published:** 2013-10-18

**Authors:** Christine E. Brown, Charles D. Warden, Renate Starr, Xutao Deng, Behnam Badie, Yate-Ching Yuan, Stephen J. Forman, Michael E. Barish

**Affiliations:** 1 Department of Cancer Immunotherapy & Tumor Immunology and Hematology & Hematopoietic Cell Transplantation, Beckman Research Institute and City of Hope National Medical Center, Duarte, California, United States of America; 2 Department of Molecular Medicine, Beckman Research Institute and City of Hope National Medical Center, Duarte, California, United States of America; 3 Department of Neurosurgery, Beckman Research Institute and City of Hope National Medical Center, Duarte, California, United States of America; 4 Department of Neurosciences, Beckman Research Institute and City of Hope National Medical Center, Duarte, California, United States of America; University of Chicago, United States of America

## Abstract

A major challenge for successful immunotherapy against glioma is the identification and characterization of validated targets. We have taken a bioinformatics approach towards understanding the biological context of IL-13 receptor α2 (IL13Rα2) expression in brain tumors, and its functional significance for patient survival. Querying multiple gene expression databases, we show that IL13Rα2 expression increases with glioma malignancy grade, and expression for high-grade tumors is bimodal, with approximately 58% of WHO grade IV gliomas over-expressing this receptor. By several measures, IL13Rα2 expression in patient samples and low-passage primary glioma lines most consistently correlates with the expression of signature genes defining mesenchymal subclass tumors and negatively correlates with proneural signature genes as defined by two studies. Positive associations were also noted with proliferative signature genes, whereas no consistent associations were found with either classical or neural signature genes. Probing the potential functional consequences of this mesenchymal association through IPA analysis suggests that IL13Rα2 expression is associated with activation of proinflammatory and immune pathways characteristic of mesenchymal subclass tumors. In addition, survival analyses indicate that IL13Rα2 over-expression is associated with poor patient prognosis, a single gene correlation ranking IL13Rα2 in the top ~1% of total gene expression probes with regard to survival association with WHO IV gliomas. This study better defines the functional consequences of IL13Rα2 expression by demonstrating association with mesenchymal signature gene expression and poor patient prognosis. It thus highlights the utility of IL13Rα2 as a therapeutic target, and helps define patient populations most likely to respond to immunotherapy in present and future clinical trials.

## Introduction

Malignant gliomas are highly aggressive and uniformly lethal human brain cancers. These tumors are histologically and molecularly diverse, exhibiting heterogeneity both between patients and within individual tumors [1,2]. This heterogeneity has confounded development of effective therapies. Recently, genomic and transcriptomic research efforts have delineated molecular subclasses of high-grade gliomas, which reflect underlying tumor biology, and predict patient outcome along with responses to therapy [3-6]. Phillips et al. [6] defined three glioblastoma subtypes based on patient prognosis and gene expression clustering: proneural, proliferative and mesenchymal. Subsequently, Verhaak et al. [5] combined genomic and expression data from The Cancer Genome Atlas (TCGA) in an unsupervised analysis to define four distinct glioblastoma subclasses: proneural, neural, classical and mesenchymal. These descriptions of tumor molecular heterogeneity provide the opportunity to evaluate potential therapeutic targets in relation to underlying tumor biology.

Some antigens are common to many gliomas and are being exploited as targets for therapeutic development. IL13Rα2 is one such antigen, expressed by a high percentage of gliomas but not at significant levels on normal brain tissue [7-10]. In IL13Rα2-expressing tumors, IL13Rα2 has been identified on stem-like malignant cells and more differentiated counterparts [11]. These features of IL13Rα2 expression make it an attractive target for brain tumor immunotherapy.

IL13Rα2 is a 42-kDa monomeric high affinity IL13 receptor distinct from the more ubiquitously expressed IL-13Rα1/IL4Rα receptor complex [12]. IL13Rα2 is proposed to down-modulate IL13Rα1/IL4Rα receptor signaling by competing for IL13 binding [13,14], and to mediate TGF-β production in monocytes and tumor-infiltrating macrophages [15,16]. Although the functional significance of IL13Rα2 expression by malignant gliomas is not well understood, it has been shown to promote tumor migration and invasion [17], and protect tumor cells from apoptosis thereby contributing to tumor growth [18].

In this study, we utilized multiple, large, publicly available datasets [5,6,19-25] to examine IL13Rα2 expression on molecularly-characterized glioma subtypes. Using this bioinformatics approach, we show that IL13Rα2 expression is positively associated with expression of mesenchymal signature genes, and conversely is negatively associated with expression of proneural signature genes [5,6]. Further we find that IL13Rα2 expression increases with glioma malignancy grade, is linked to activated immune pathways by IPA analysis, and is a prognostic indicator of poor patient survival. These observations enhance understanding of IL13Rα2 as a therapeutic target and provide guidance for the design of patient specific therapies tailored for optimal therapeutic responses.

## Methods

### Ethics Statement

Primary glioma specimens used to generate patient glioma cell lines (PBTs) were obtained in accordance with City of Hope Institutional Review Board-approved protocols. All other patient information was obtained from publicly available datasets (Table 1).

**Table 1:** Publicly Available Patient Data Sets Used for Gene Expression Profiling and Prospective Analyses.

Study	Source	Sample Size	Grade	Age (mean±SD)	Gender
Freije [19]	GEO; (GSE4412)	74 primary	I: 0; II: 0; III: 24; IV: 50	21.5 ± 20.1	Male: 28; Female: 46
Gravendeel [20]	GEO; (GSE16011)	276 primary; 8 normal	I: 8; II: 24; III: 85; IV: 159	51.5 ± 14.7	Male: 184; Female: 92
Lee [21]	GEO; (GSE13041)	191 primary	I: 0; II: 0; III: 0; IV: 191	53.8 ± 13.6	Male: 117; Female: 74
Murat [22]	GEO; (GSE7696)	70 primary; 11 recurrent; 4 normal	I: 0; II: 0; III: 0; IV: 70	51.0 ± 9.2	Male: 51; Female: 19
Petalidis [23]	GEO; (GSE1993)	54 primary; 11 recurrent	I: 0; II: 6; III: 13; IV: 35	49.9 ± 17.9	Male: 36; Female: 18
Phillips [6]	GEO; (GSE4271)	77 primary; 23 recurrent	I: 0; II: 0; III: 21; IV: 56	45.5 ± 13.0	Male: 52; Female: 25
Sun [24]	GEO; (GSE4290)	157 primary; 23 normal	I: 0; II: 57; III: 41; IV: 59	50.9 ± 16.2	Male: 63; Female: 46
TCGA [25]	TCGA Data Portal; (11/29/2010)	354 primary	I: 0; II: 0; III: 0; IV: 339	56.6 ± 14.5	Male: 211; Female: 128

### Evaluation of Patient-Derived Cell Lines

#### Cell Lines

Primary glioma cell lines (PBT) were derived from patients undergoing tumor resections at City of Hope [11,26]. In some cases tumor explants were expanded by heterotopic subcutaneous (s.c.) passaging in mice prior to growth and characterization in culture [26], and in such cases the s.c. passage number is reported after the PBT number (i.e. PBT003-4 and PBT017-4 were s.c. passaged four times). PBT lines were grown in neural stem cell media (DMEM:F12 (Irvine Scientific); 1:50 B27 (Invitrogen, Carlsbad, CA); 5 µg/mL heparin (Abraxis Pharmaceutical Products, Schaumburg, IL), 2 mM L-glutamine (Irvine Scientific) [27,28] , supplemented with 20 ng/mL EGF (R&D Systems, Minneapolis, MN) and 20 ng/mL bFGF (R&D Systems) twice a week. U251T glioblastoma adherent cells (gift from Dr. Waldemar Debinski, Wake Forest School of Medicine) were grown in DMEM (Irvine Scientific) supplemented with 10% FCS, 2 mM L-glutamine, and 25 mM HEPES.

#### Flow cytometry

 Cell-surface phenotypes were assayed as previously described [29] using either goat polyclonal anti-IL13Rα2 (AF146, R&D Systems, Minneapolis, MN) followed by mouse anti-goat fluorescein isothiocyanate (FITC) (Jackson ImmunoResearch, West Gove, PA), or phycoerythrin (PE)-conjugated mouse anti-human CD133/1 and anti-human CD133/2 (Miltenyi Biotec, Bergisch Gladbach, Germany), CD44 (clone 515; BD Biosciences) or CD54/ICAM-1 (clone 1A29; BD Biosciences). Percent of immunoreactive cells was calculated using the subtraction method (FCS Express version 3 software; De Novo Software, Los Angeles, CA). Statistical analysis was calculated by student t-test of the log transformed MFI values.

#### Gene expression profiling in cell lines

Total cellular RNA was extracted using the Qiagen RNeasy Kit (Qiagen), and quality checked using Agilent Bioanalyzer nano 6000 chip. Triplicate samples (except duplicate for U251T) were hybridized to Affymetrix HG-U133 Plus 2.0 arrays following standard Affymetrix procedures and the Affymetrix 3’ Express Kit, Expression values were RMA normalized using Partek^®^ Genomics Suite^TM^ (Partek, Inc., St. Louis, MO) and averaged among triplicate samples (duplicate for U251T). Microarray data is available in GEO data series GSE40904.

### Evaluation of Patient Datasets

Patient data for bioinformatic analyses was drawn from a cohort of eight publicly available datasets (Table 1) [6,19-25] from which metadata was downloaded on or before 11/29/2010 as part of a biomarker database developed at City of Hope (BRAVO database [30]). For the TCGA dataset, samples have been added since our initial download, and for this reason the numbers of samples are fewer than presently available.

#### Differential IL13Rα2 Expression based on Tumor Grade and Histological Subtype

Data describing IL13Rα2 gene expression for WHO tumor grades I-IV and histological subtype were for 429 primary patient samples taken from combined Sun et al. [24] and Gravendeel et al. [20] data sets, and evaluated by 2-way ANOVA with appropriate linear contrast was used to compare data sets using Partek^®^ Genomics Suite^TM^. Fold-change values were calculated based upon the least-squares mean using Partek^®^ Genomics Suite^TM^. Prior to statistical analysis, data were normalized using robust multichip average (RMA) normalization [31]. 

#### Model of Bimodal IL13Rα2 Expression

 Estimated frequencies of IL13Rα2 over-expression for each array platform were derived from a bimodal distribution model. For each platform, the density distribution was first calculated using the ‘density’ function in R [32], followed by fitting parameters for mixture mode (Equation 1) using the ‘nls’ function in R [32]:

$$Density=\left(p\right)\left(\frac{1}{\sqrt{2\pi \sigma^{2}}}\right)\left(e^{\frac{-{\left(x-\mu_{1}\right)}^{2}}{2\sigma^{}}}\right)+\left(1-p\right)\left(\frac{1}{\sqrt{2\pi \sigma^{2}}}\right)\left(e^{\frac{-{\left(x-\mu_{2}\right)}^{2}}{2\sigma^{}}}\right)$$

This model describes the relationship between the expression level, *x*, and the corresponding frequency in the density plot. In this model, *p* = the frequency of tumors over-expressing IL13Rα2, μ_1_ = mean expression of the IL13Rα2-positive population, μ_2_ = mean expression of the IL13Rα2-negative population, and σ = the standard deviation (identical for both distributions). The measured variance was roughly equal for the IL13Rα2-high and IL13Rα2-low expressing populations, and using an equal variance for both distributions increased the accuracy of the estimate for *p*. However, a better fit was obtained for the data from Pedalidis et al. [8], using an alternative model incorporating two different standard deviations (σ_1_ = the standard deviation of the IL13Rα2-positive population and σ_2_ = the standard deviation of the IL13Rα2-negative population) (Equation 2):

$$Density=\left(p\right)\left(\frac{1}{\sqrt{2\pi \sigma_{1}{}^{2}}}\right)\left(e^{\frac{-{\left(x-\mu_{1}\right)}^{2}}{2\sigma_{1}{}^{}}}\right)+\left(1-p\right)\left(\frac{1}{\sqrt{2\pi \sigma_{2}{}^{2}}}\right)\left(e^{\frac{-{\left(x-\mu_{2}\right)}^{2}}{2\sigma_{2}{}^{}}}\right)$$

#### Principal Component Analysis

**Figure 2:**
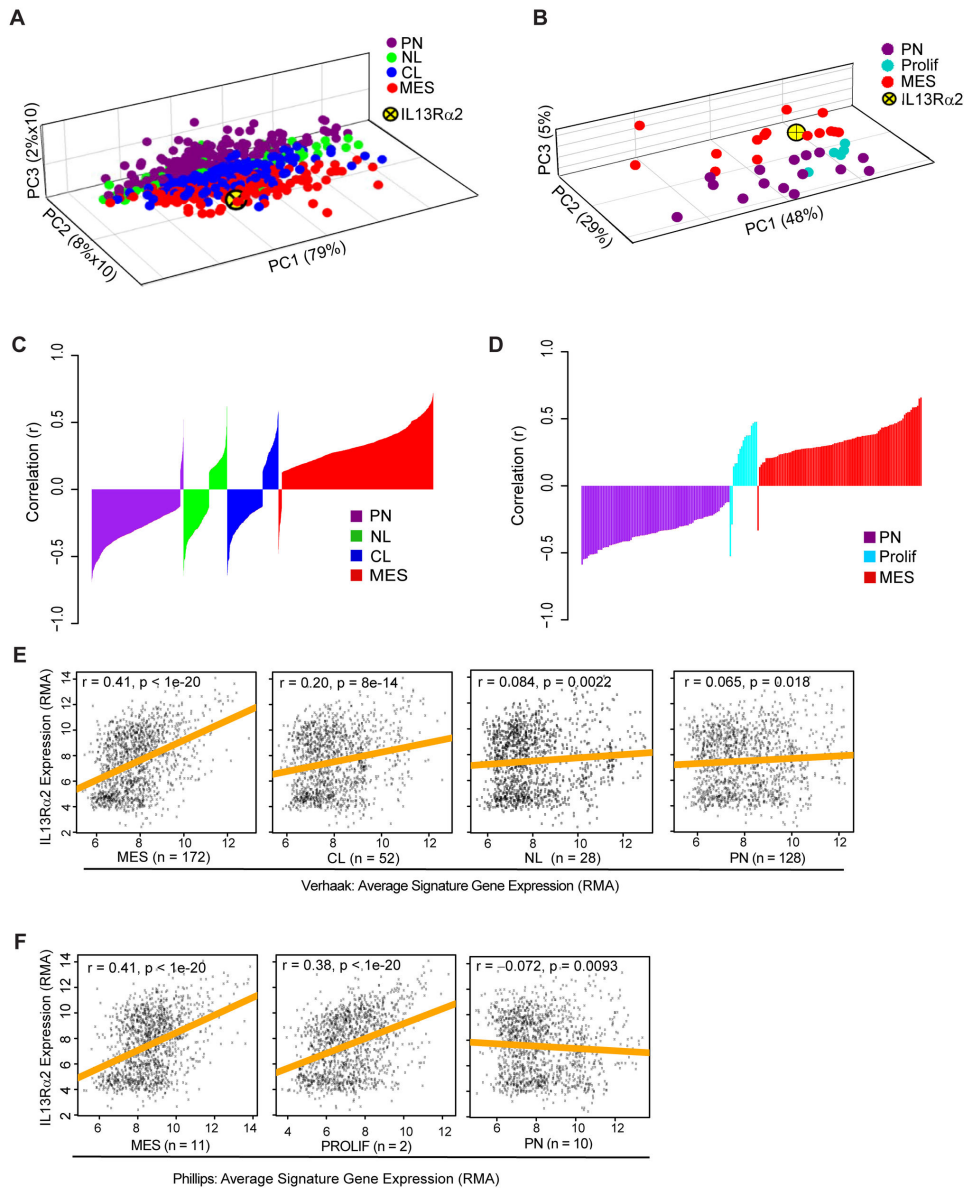
IL13Rα2 expression is associated with mesenchymal signature gene expression in gliomas. (A, B) Principal Component Analysis (PCA) plots of signature genes with respect to IL13Rα2 for glioma subclasses defined by (A) Verhaak et al. [5] (proneural (PN), neural (NL), classical (CL) and mesenchymal (MES)) and (B) Phillips et al. [6] (proneural (PN), proliferative (PROLIF) and mesenchymal (MES). Each point represents the position of one signature gene. (C, D) Silhouette plots of Pearson correlation coefficients for all significant correlations (FDR < 0.05) between IL13Rα2 and probes for genes defining glioma subtypes as defined by (C) Verhaak et al. [5], and (D) Phillips et al. [6] . Correlations are sorted based upon GBM subtype and then ordered by increasing correlation coefficient values. (E-F) Scatter plots for IL13Rα2 expression versus the average expression of (E) TCGA mesenchymal (MES), proneural (PN), classical (CL) and neural (NL) genes, and (F) the average expression of Phillips mesenchymal (MES), proliferative (PROLIF), and proneural (PN) genes). Linear regression of IL13Rα2 expression as a function of gene expression is shown by the orange lines. Number of signature genes (n) used to calculate average signature gene expression is reported for each plot (Methods and Datasets S3 and S4 in File S2).

PCA was performed in Partek^®^ Genomics Suite^TM^, with a correlation dispersion matrix and normalized eigenvector scaling. Gene expression data from Sun et al. [24] and Gravendeel et al. [20] was used to define principal components that delineate the greatest variation in tumor IL13Rα2 expression for the 685 and 35 signature genes used to define the molecular subtypes of Verhaak et al. and Phillips et al., respectively [5,6]. Accordingly, the PCA plots (Figures 2A and 2B) present variation in expression between tumors where each dot represents a signature gene.

#### Correlations with Signature Gene Expression (Silhouette plots)

 Correlation analyses were performed on expression data from high-grade (WHO Grade III and IV) brain tumors (Table 1), and correlations were calculated per probe per array per cohort. Analyses were conducted separately for each probe in each study for the 685 genes classifying proneural, neural, classical, and mesenchymal subtypes of Verhaak et al. [5] (Dataset S1), and for the 35 genes classifying proneural, proliferative, and mesenchymal subtypes of Phillips et al. [6] (Dataset S2). Pearson correlation coefficients and the corresponding correlation test were calculated using R [32], and considered significant at False Discovery Rate (FDR) < 0.05 (calculated by the method of Benjamini and Hochberg [33]).

#### Survival Analyses

**Figure 1:**
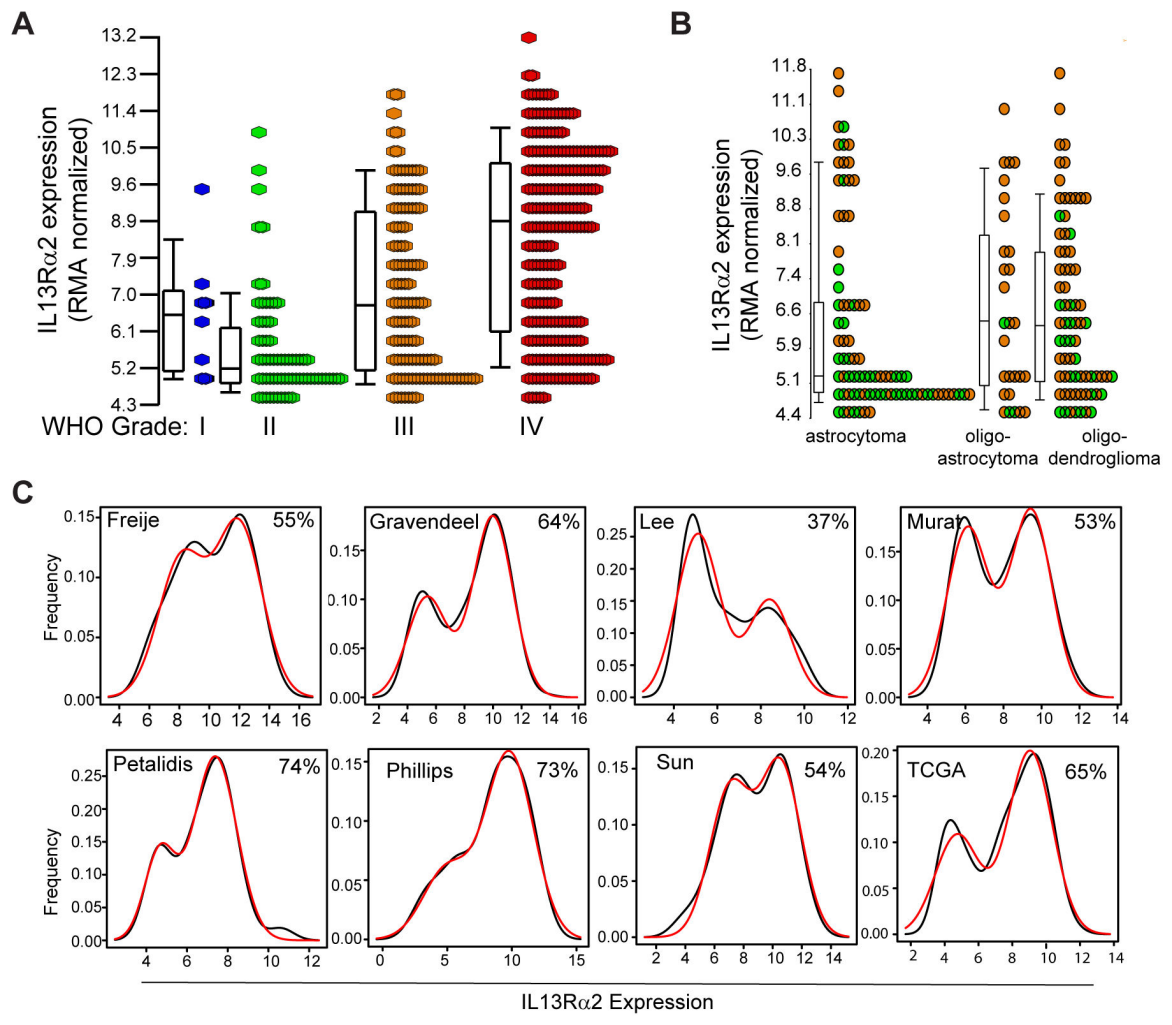
Expression of glioma IL13Rα2 with respect to tumor grade and histological sub-type. (A) Comparison of IL13Rα2 gene expression levels (robust multichip average, RMA) versus glioma tumor grade (WHO grades I-IV) from a combined data set of 429 patient samples [20,24]. The level of IL13Rα2 expression is 3.5-fold higher for WHO grade IV patients as compared to all other patients (2-way ANOVA with linear contrast, p=1.5 x 10^-9^). Shown are box plots marking median (bar), 25^th^ and 75^th^ percentiles (box), and 10^th^ and 90^th^ percentiles (error bars). (B) Comparison of IL13Rα2 gene expression levels for 184 samples [20,24] segregated based on glioma WHO grade II (orange circles) and grade III (green circles), and histological sub-types: astrocytoma, oligoastrocytoma and oligodenroglioma. No significant difference in IL13Rα2 expression levels were observed for the three patient cohorts (p=0.83, 2-way ANOVA). Box plots define percentiles as in (A). (C ) Biomodal distributions of IL13Rα2 for GBM (WHO IV) as defined by eight gene-array studies [5,6,19-25] (black line). Reported for each study is the frequency of tumors over-expressing IL13Rα2 estimated using a non-linear least squares regression (red line) .

Data for survival analysis were aggregated from eight studies [6,19-25] (Table 1). IL13Rα2, over-expression was determined using our NLS model (Figure 1C), and survival differences between ’high‘ and ’low‘ expression groups were visualized by Kaplan-Meier plots and compared using Cox regression analysis, with p-values calculated by log-rank test using the Survival package in R [32]. For mesenchymal and proneural subsets, ’high’ and ’low‘ groups were segregated based on median expression values. For genome-wide survival analysis (Dataset S7 in File S2), Affymetrix probes present in all eight cohorts were used, and  ’high’ and ’low‘ groups were defined based on median expression levels per cohort (including IL13Rα2).  A log-rank test was used to calculate p-values for survival across all eight cohorts.

### Definition of Gene Probes to Represent Glioma Molecular Subtypes

Averaged expression values were calculated using a single Affymetrix array per cohort: the HG-U133A for Freije et al. [19], Phillips et al. [6], Lee et al. [21], and Petalidis et al. [23]; the HG-U133 Plus 2.0 array for Sun et al. [24], Gravendeel et al. [20], and Murat et al. [22]; and the HT HG-U133A array for the TCGA cohort [25]. 

**Figure 3:**
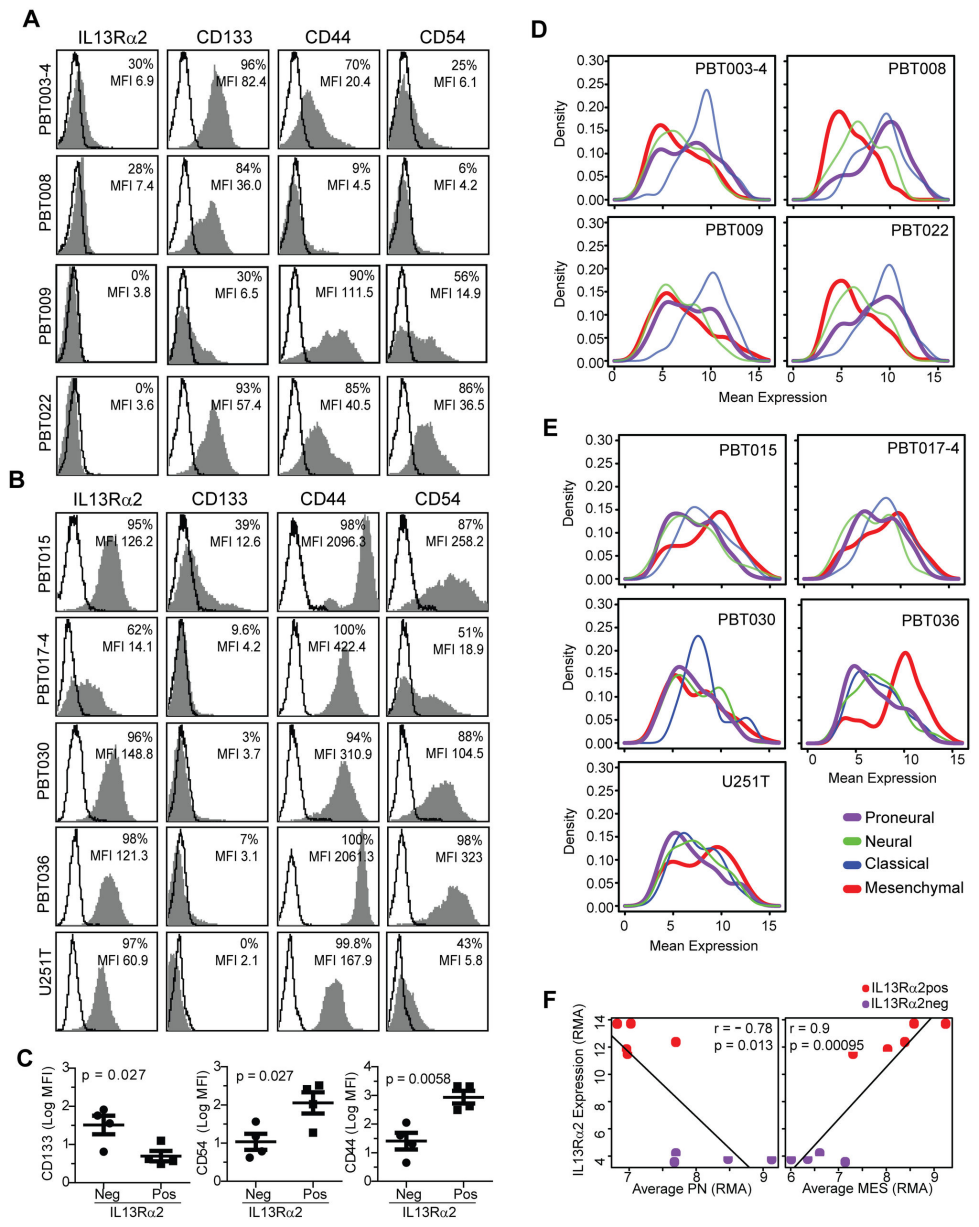
IL13Rα2 expression is associated with mesenchymal gene expression in low-passage primary glioma cell lines. Flow cytometry analyses of (A) IL13Rα2-negative glioma cell lines (PBT003-4, PBT008, PBT009, PBT022), and (B) IL13Rα2-positive glioma cell lines (PBT015, PBT017-4, PBT030, PBT036, and U251T) for expression of the proneural marker CD133 and mesenchymal markers CD44 and CD54/ICAM-1 (grey histograms). Solid lines show isotype and secondary control antibodies. (C) Comparison of mean fluorescence intensity (MFI) of IL13Rα2-negative and IL13Rα2-positive glioma cell lines shown in panels A and B. (D-E) Distribution plots of Affymetrix gene array expression analyses of (D) IL13Rα2-negative and (E) IL13Rα2-positive glioma cell lines for mesenchymal, classical, neural and proneural signature gene expression. (F) Correlation of mesenchymal (MES) versus proneural (PN) average gene expression with RMA normalized IL13Rα2 expression in glioma cell lines.

In order to determine the optimal Affymetrix probes (present in all 8 cohorts) to most accurately reflect the four TCGA subtypes (PN, NL, CL and MES), we evaluated signature gene probes on the HT HG-U133A array and used the Verhaak et al. [5] training dataset to calculate the fold-change, p-values, and FDR values for each probe. Fold-change values were calculated on a linear scale by comparing expression in tumors of the same subtype in the training subset with its expression in the other subtypes. For example, the ratio for mesenchymal patient expression was [MES expression] / [PN expression + NL expression + CL expression]. If the subtype ratio was greater than 1, then the fold-change equaled the ratio. If the subtype ratio was less than 1, the fold-change was equal to the negative inverse of the subtype ratio. FDR values were calculated using the method of Benjamini and Hochberg [33] based on the distribution of t-test p-values. A probe was validated for association with a signature gene if its fold-change value was > 2 and the FDR < 0.05, and validated probes with associated subtypes are listed in Dataset S3. Expression levels for each of these probes were averaged to obtain an overall index of subtype signature gene expression as used in Figures 2E and 3F, and used to construct the histograms in Figure 3D and 3E. 

For average gene expression of the subtypes defined by Phillips et al. [6] (Figure 2F) signature gene probes were included in the analysis for those Affymetrix probes on the HT HG-U133A, but not the HT HG-U133B array, since only the U133A probes were common to all 8 cohorts (Dataset S4). 

### Systems-Level Pathway Analyses

 Statistical enrichments for canonical pathways defined by IPA (Ingenuity® Systems, www.ingenuity.com) were calculated using Fisher’s Exact test. Pathway enrichments for genes showing positive correlations with IL13Rα2 expression in patient tumors were defined for genes displaying at least 4 of 8 significant correlations [6,19-25]. For genes identified from Affymetrix arrays as differentially expressed in glioma cell lines (IL13Rα2-positive versus IL13Rα2-negative), correlations were considered statistically significant at FDR < 0.05 (Benjamini and Hochberg [33]). Pathways for the TCGA signature genes were based on the mesenchymal (n=216), classical (n=162), neural (n=129), proneural (n=178) signature genes defined by Verhaak et al. [5].

## Results

### Glioma IL13Rα2 is Associated with Increased Malignancy Grade

IL13Rα2 expression was evaluated with respect to tumor malignancy grade using a data set combined from Sun et al. [24] and Gravendeel et al. [20], the two largest studies including World Health Organization (WHO) grades I-IV tumors (Table 1). We find that IL13Rα2 levels are 2.8-fold higher in high-grade (WHO III and IV) than in low-grade (WHO I and II) tumors, a highly statistically significant difference even after factoring out potential batch effects (2-way ANOVA with linear contrast, p=2.4 x 10^-4^) (Figure 1A). This trend is even stronger (3.5-fold expression increase) when WHO grade IV glioblastoma (GBM) are compared to all other tumor grades (2-way ANOVA with linear contrast, p=1.5 x 10^-9^). Additionally, within high-grade gliomas (WHO III and IV), IL13Rα2 expression also increases with malignancy grade, with a 2.5-fold increase for WHO grade IV versus grade III tumors (p=2.9 x 10^-8^). These results expand upon previous immunochemical studies evaluating a smaller tumor sample set showing a similar increase in the frequency of glioma IL13Rα2 expression with tumor grade [34]. 

Since GBM (defined as astrocytomas WHO grade IV) displayed the highest levels of IL13Rα2 expression, we tested whether astrocytomas (WHO II and III) over-expressed IL13Rα2 more frequently than histologically distinct oligoastrocytomas or oligodendrogliomas of the same grade (WHO II and III). Astrocytomas did not exhibit a statistically significant difference in IL13Rα2 expression from oligoastrocytomas and oligodendrogliomas (p=0.69, 2-way ANOVA with linear contrast to account for batch effects due to study) (Figure 1B), and no discernible differences were observed between the three histological subtypes (p=0.83, 2-way ANOVA to account for batch effects due to study). We also examined a possible relationship between IL13Rα2 expression and tumor recurrence (Figure S1 in File S1), and did not find significant differences between primary and recurrent tumors (0.18 ≤ p ≤ 0.35) in the 3 cohorts examined [6,22,23]. These results indicate that IL13Rα2 expression is associated with glioma malignancy grade, and is independent of histological subtype and tumor recurrence.

For the high-grade gliomas analyzed by Sun [24] and Gravendeel [20], our analysis reveals a clear bimodal distribution of IL13Rα2 expression for the high-grade gliomas that is particularly evident for GBM (WHO IV) specimens (Figure 1A), as also noted by Jarboe et al. [8]. This bimodal IL13Rα2 distribution was subsequently confirmed for an expanded data set encompassing eight independent gene-array studies with a total of 959 GBM samples [5,6,19-25] (Figure 1C, Table 1). Using these data, we estimated IL13Rα2 over-expression frequency between cohorts by modeling IL13Rα2 expression as the sum of two normal distributions. The percentage over-expression in 7 of 8 cohorts was 53-73% (except Lee et al. [21] at 37%) (Figure 1C, Table S1 in File S1). Within these multiple, large datasets, the average IL13Rα2 over-expression frequency is estimated to be 58% for GBMs, which is higher than reported by Jarboe et al. [8] for a smaller sample set. 

### Association of Glioma IL13Rα2 with Mesenchymal Signature Gene Expression

We explored the possibility that biomodal expression of IL13Rα2 may reflect associations with the distinct molecular subtypes identified for GBMs. In one approach, Principal Component Analysis (PCA) was used to evaluate potential associations of IL13Rα2 with expression of signature genes identified by Verhaak et al. [5] and Phillips et al. [6] to distinguish between subclasses of high-grade gliomas. Considering a cohort of 428 tumors (WHO Grade I-IV) compiled from Sun et al. [24] and Gravendeel et al. [20], we find that tumor expression of IL13Rα2 is most closely associated with expression of the mesenchymal signature gene sets as defined by both Verhaak and Phillips [5,6] (Figure 2A, 2B and Table S2 in File S1).

In a related analysis, we evaluated correlations between IL13Rα2 expression and the signature genes identified by Verhaak [5] and Phillips [6] for a larger sample of 1143 high-grade tumors from eight independent gene expression studies [6,19-25]. The silhouette plots in Figure 2C and 2D display all significant correlation coefficients (FDR < 0.05) grouped by GBM subclass. Correlation calculations were independently computed for each gene probe per array per study (eight GBM cohorts) [6,19-25], so each gene is typically represented more than once. This probe-level approach was validated for test genes representative of mesenchymal (CHI3L1), proneural (DLL3) and classical (EGFR) subtypes (Figure S2 in File S1). While many probes for these test genes showed non-significant correlations, the overall pattern of significant correlations were congruent with their assigned subtypes (Figure S2 in File S1). For IL13Rα2 many probes also showed non-significant correlations (Figure S2 in File S1), however similar to the mesenchymal test gene CHI3L1, IL13Rα2 strongly correlated with mesenchymal signature genes of both Verhaak [5] and Phillips [6] with the majority of mesenchymal probes showing at least one significant positive correlation (FDR < 0.05) (Verhaak: 171 of 215 genes, 79.5% of genes; Phillips: 12 of 15 genes, 80% of genes) (Figure 2C, 2D and Table S3 in File S1; see Datasets S1 and S2 in File S2 for comprehensive lists). IL13Rα2 expression also showed a positive correlation with the majority of proliferative signature genes of Phillips [6] (4 of 5 genes, 80% of genes). Reciprocally, the majority of significant probe correlations with proneural gene signature genes defined by Verhaak [5] and Phillips [6] showed at least one negative correlation (Verhaak: 132 of 178 genes, 74.2% of genes; Phillips: 13 of 14 genes, 92.9% of genes) (Figure 2C, 2D and Table S3 in File S1; see Datasets S1 and S2 in File S2 for a comprehensive list). Glioma IL13Rα2 expression did not display consistent correlation patterns with genes defining the neural and classical subclasses of Verhaak [5] (Figure 2C and 2D, and Table S3 in File S1). 

In a third independent but related analysis, IL13Rα2 expression was compared to average subtype signature gene expression of Verhaak [5] and Phillips [6] for 1318 patient tumors from 8 studies [6,19-25] . The goal of this analysis was to test tumor associations independently of prior tumor subtype classification. For this analysis we selected one array per study requiring a gene probe to be represented on each of the arrays chosen, as well as meeting our expression criteria (see Methods, Datasets S3 and S4 in File S2). Consistent with the PCA and silhouette analyses, IL13Rα2 expression shows the strongest positive correlation with average tumor mesenchymal signature gene expression (Verhaak: r=0.41, p<10^-20^; Phillips: r=0.41, p<10^-20^), and shows almost no or negative correlation with average tumor proneural signature gene expression (Verhaak: r=0.065, p=0.018; Phillips: r= −0.072; r=0.0093) (Figure 2E and 2F). While the strength of this mesenchymal correlation varied between studies (0.18 < r < 0.46, Figure S3 in File S1), the correlation was always statistically significant. Also consistent with the silhouette analysis, IL13Rα2 expression positively correlates with a subset of proliferative signature genes (2 of 5 genes: HMMR and DTL) (Phillips: r=0.38, p<10^-20^). The other 3 proliferative genes could not be included in this analysis because they were not evaluated in all 8 studies (see Methods, Dataset S4). Weaker positive correlations were observed for the classical and neural signature genes (Verhaak: CL, r=0.20, p=8^-14^; NL, r=0.084, p=0.0022). Consistent with these findings, for the limited set of tumors with reported subtype classification (Verhaak training set n=161; Phillips n=77), mesenchymal tumors also express higher levels of IL13Rα2 (1.68-fold; p = 0.00015 for Verhaak [6]; 2.44-fold, p = 0.028 for Phillips [5]) than do proneural tumors (Figure S4 in File S1). In this limited tumor dataset, IL13Rα2 is also highly over-expressed in the proliferative subtype of Phillips [5], and the neural subtype of Verhaak [6] (Figure S4 in File S1).

It is significant to note that the positive correlation of IL13Rα2 expression with average mesenchymal signature gene expression and the negative correlation with proneural signature gene expression is consistent across two independent studies that defined GBM subtypes based on different criteria — patient survival for Phillips et al. [6] and unsupervised clustering for Verhaak et al. [5] — and our findings are similar for both studies despite the relatively small number of genes common to both subtype-defining signature gene sets. Only three mesenchymal signature genes (CHI3L1/YKL40, SERPINE1 and TIMP1), and five proneural signature genes (DLL3, KLRC3, SCG3, C20orf42, and NCAM1) are common to both the Verhaak et al. [5] and Phillips et al. [6] definitions, and none of the five proliferative signature genes of Phillips [6] are included in any of the subclasses defined by Verhaak [5]. Our results thus persist independently of the specific genes defining each subclass, and therefore suggest that IL13Rα2 expression may be closely related to fundamental biological differences between mesenchymal and proneural GBM subtypes. 

Interestingly, in parallel with what is seen for IL13Rα2, mesenchymal signature genes are also up-regulated in high-grade gliomas (WHO IV vs. III/II/I: 93 of 211 signature genes; 44.1%) (Figure S6B in File S1), and proneural signature genes are commonly down-regulated in high-grade tumors (WHO IV vs. III/II/I: 59 of 171 signature genes; 34.5%) (Figure S6B in File S1). These findings again suggest an underlying biological basis for the common expression profiles of mesenchymal signature genes and IL13Rα2.

Taken together, our data demonstrates using three distinct approaches (Figure 2) that IL13Rα2 expression is most strongly and consistently associated with mesenchymal signature gene expression. It should be noted, however, that IL13Rα2 expression is not restricted to mesenchymal subclass tumors, and IL13Rα2 expression also consistently correlates with the proliferative signature genes of Phillips [6]. While the association between IL13Rα2 and proliferative signature genes is intriguing, this was difficult to assess further because of the limited set of five genes defining the proliferative tumor subtype, and for technical reasons three could not be evaluated in all of the eight studies queried for this analysis (see Methods). It should be further noted that IL13Rα2 was not originally identified as a mesenchymal signature gene, and this association may not be sufficiently strong to serve as a signature gene. In comparison to other mesenchymal signature genes, IL13Rα2 does not typically show as strong an association with either the average mesenchymal gene correlation (Figure S5 in File S1) or the differences in expression between mesenchymal and proneural subclass tumors (Figure S6A in File S1). Nevertheless, these three distinct approaches employing the subtype definitions of two independent studies [5,6] (Figure 2) clearly show significant and consistent associations between IL13Rα2 and mesenchymal signature gene expression, and as such suggest functional consequences for the basic biology of these tumors. 

Moving forward, since no significant differences were observed in the association of IL13Rα2 with mesenchymal signature gene expression as defined by Phillips et al. [6] or by Verhaak et al. [5], we focused on the classifications defined by Verhaak et al. [5] because the greater number of signature genes differentiating each subclass enabled probing the potential biological significance of this interaction. 

### Mesenchymal Signature Gene Expression Correlates with High IL13Rα2 Expression in Patient-derived Glioma Cell Lines

A panel of low-passage primary glioma cell lines [11,26] were used to evaluate whether our findings extend to more homogeneous populations of malignant GBM cells. IL13Rα2-positive (n=4) and -negative (n=4) glioma lines derived either from freshly dispersed human tumor specimens or after heterotopic passaging of patient tumor tissue in immunodeficient mice [4,26], along with the established glioma line U251T, were assessed for expression of mesenchymal and proneural signature genes. By flow cytometry, the IL13Rα2-positive glioma cell lines (PBT015, PBT017-4, PBT030, PBT036, U251T) generally express higher levels of the mesenchymal adhesion markers CD44 and CD54/ICAM-1 than the IL13Rα2-negative glioma lines (PBT003-4, PBT008, PBT009, PBT022) (Figure 3A-3C). Moreover, the IL13Rα2-positive lines frequently display lower CD133 expression than do the IL13Rα2-negative lines (Figure 3A-3C), consistent with previous reports that high CD133 expression by glioma cell lines grown in neural stem cell media is indicative of a proneural phenotype [35,36].

 Affymetrix expression profiling was then used to test in greater detail the association between mesenchymal phenotypes and IL13Rα2 expression in these nine GBM cell lines. As shown in Figure 3D and 3E, IL13Rα2-negative cell lines express proneural signature genes at higher levels than mesenchymal genes, and reciprocally IL13Rα2-positive cell lines express mesenchymal signature genes at higher levels than proneural genes. When comparing average TCGA subtype gene expression levels, mesenchymal signature gene expression positively correlates most strongly with L13Rα2 expression for each cell line (Figure 3F, r=0.90, p=0.00095), whereas average proneural gene expression show the strongest negative correlation (Figure 3F, r= -0.78, p=0.013). Although the cell lines express varying levels of classical and neural signature genes (Figure 3D and 3E), strong mesenchymal gene expression was always associated with high IL13Rα2 expression in the primary cell lines (Figure 3F).

### Systems-Level Analysis of Genes Coexpressed with IL13Rα2

Ingenuity Pathway Analysis (IPA) was used to assess the potential biological significance of IL13Rα2 expression in GBMs. In an unsupervised approach, we first defined two lists: genes positively correlated with IL13Rα2 expression in at least four of the eight patient cohorts (all correlations shown in Dataset S5; FDR <0.05), and genes differentially expressed in IL13Rα2-positive versus IL13Rα2-negative patient-derived cell lines (Dataset S6; |fold-change| > 1.5; FDR <0.05). We then used IPA analysis to identify functional pathways associated with these two IL13Rα2-linked gene sets, along with pathways associated with signature genes of Verhaak et al. [5] for mesenchymal, classical and proneural subclasses (note that no pathways with FDR < 0.05 were assigned by IPA to neural signature genes). A matrix of the total numbers of pathways identified by IPA analysis with FDR <0.05 and overlapping between different subclasses is presented in Table S4 in File S1. More detailed examination showed that many IL13Rα2-associated pathways are enriched in both patient samples and cell lines [31% (14 of 45) for patient samples; and 61% (14 of 23) for cell lines; Tables S5 and S6 in File S1]. Consistent with our previous findings, pathways associated with over-expression of IL13Rα2 in patient samples and patient-derived cell lines most frequently overlapped with canonical pathways associated with mesenchymal signature genes, and not those associated with proneural signature genes. Further, our analysis indicates that for these two IL13Rα2-associated gene sets, immune-related pathways are among the most enriched [44% (20 of 45) for patient samples and 48% (11 of 23) for cell lines; Tables S5 and S6 in File S1], and that eight immune-related pathways are among those common to the patient samples and cell lines (Tables S5 and S6 in File S1). As shown in Table 2 (excerpted from Tables S5-S7 in File S1), TREM1 Signaling, Antigen Presentation, and Hepatic Fibrosis/Hepatic Stellate Cell Activation were among the eight most enriched pathways in both datasets. 

**Table 2:** IPA Pathways Associated With IL13Rα2 Expression in Patient Tumor Samples, IL13Rα2-positive Cell Lines, and Mesenchymal Signature Genes.

**Study**	**Source**	**Sample Size**	**Grade**	**Age (mean±SD)**	**Gender**
Freije [19]	GEO; (GSE4412)	74 primary	I: 0; II: 0; III: 24; IV: 50	21.5 ± 20.1	Male: 28; Female: 46
Gravendeel [20]	GEO; (GSE16011)	276 primary; 8 normal	I: 8; II: 24; III: 85; IV: 159	51.5 ± 14.7	Male: 184; Female: 92
Lee [21]	GEO; (GSE13041)	191 primary	I: 0; II: 0; III: 0; IV: 191	53.8 ± 13.6	Male: 117; Female: 74
Murat [22]	GEO; (GSE7696)	70 primary; 11 recurrent; 4 normal	I: 0; II: 0; III: 0; IV: 70	51.0 ± 9.2	Male: 51; Female: 19
Petalidis [23]	GEO; (GSE1993)	54 primary; 11 recurrent	I: 0; II: 6; III: 13; IV: 35	49.9 ± 17.9	Male: 36; Female: 18
Phillips [6]	GEO; (GSE4271)	77 primary; 23 recurrent	I: 0; II: 0; III: 21; IV: 56	45.5 ± 13.0	Male: 52; Female: 25
Sun [24]	GEO; (GSE4290)	157 primary; 23 normal	I: 0; II: 57; III: 41; IV: 59	50.9 ± 16.2	Male: 63; Female: 46
TCGA [25]	TCGA Data Portal; (11/29/2010)	354 primary	I: 0; II: 0; III: 0; IV: 339	56.6 ± 14.5	Male: 211; Female: 128

FDR < 0.05; Immune function-related pathways are indicated in bold.

^1^ IPA pathways associated with differential gene expression between IL13Rα2-positive and IL13Rα2-negative cell lines (|fold-change| > 1.5; FDR <0.05).

^2^ IPA pathways associated with differential gene expression for patient samples, define as genes positively correlated with IL13Rα2 in at least 4 independent cohorts.

^3^ IPA pathways associated with canonical mesenchymal signature genes defined by Verhaak et al. [5]. Note that Hepatic Fibrosis and TREM1 signaling are among the top 8 enriched pathways for all 3 gene lists.

As was seen for pathways associated with IL13Rα2 expression in patient samples and cell lines, many (41%; 29 of 71) pathways associated with mesenchymal signature gene expression [5] were also immune-related (Table S7 in File S1). In addition, pathways associated with over-expression of IL13Rα2 in patient samples and cell lines were among the canonical pathways associated with mesenchymal signature genes [5] (9 of 71, 13%, for both patients and cell lines, with an additional 6 for cell lines only (total of 15 of 71 or 21%; Table S7 in File S1). Further, of the nine pathways associated with all three gene sets, five are associated with immune system functions (Table 3). Of these, Hepatic Fibrosis and TREM1 Signaling are particularly highly correlated. Other immune-related pathways including IL-6 and HMGB, along with NF-κB signaling, showed qualitatively similar enrichments although falling just short of the FDR < 0.05 criterion in one of the three groups (Figure S7 in File S1). In contrast, only one pathway associated with classical-subtype signature genes was common to IL13Rα2-enriched patient samples and cell lines (Table S8 in File S1). Notably, this single pathway is immune-related. No IPA-defined pathways for proneural signature genes were shared with IL13Rα2 (or were immune related; Table S9 in File S1), consistent with the antiphase correlation with IL13Rα2 expression shown by signature genes of the mesenchymal and proneural tumor sublasses (Figure 2C). The high associations of IL13Rα2-related pathways as defined by IPA analysis with immune system functions are consistent with previous reports of elevated immune activation in mesenchymal-subtype tumors [37], and suggests that IL13Rα2 over-expression in other glioma subtypes (Figure S4 in File S1) may be an indicator of on-going immune processes in these tumors as well. 

**Table 3:** Overlapping IPA Pathways Associated with Expression of Mesenchymal Signature Genes and Genes Up-regulated in IL13Rα2-positive Cell Lines and IL13Rα2 Over-expressing Patient Samples.

IPA-defined Pathway*	**Mesenchyma**l **Signature Genes** (**FDR**)	**IL13Rα2-positive Cell Lines (FDR)**	**IL13Rα2 Over-expressing Patient Samples (FDR)**
**Hepatic Fibrosis / Hepatic Stellate Cell Activation**	0.0000054	0.00021	0.0012
**TREM1 Signaling**	0.000072	0.0036	0.0021
**Dendritic Cell Maturation**	0.000079	0.049	0.020
Integrin Signaling	0.00096	0.0068	0.014
Agrin Interactions at Neuromuscular Junction	0.0033	0.045	0.031
Caveolar-mediated Endocytosis Signaling	0.016	0.00062	0.012
**Type I Diabetes Mellitus Signaling**	0.016	0.0091	0.0036
Death Receptor Signaling	0.030	0.0036	0.012
**Role of Macrophages, Fibroblasts and Endothelial Cells in Rheumatoid Arthritis**	0.042	0.034	0.019

* Top overlapping IPA pathways with FDR < 0.05. Immune function-related pathways are indicated in bold.

### Expression of IL13Rα2 and Mesenchymal Signature Genes are Markers of Poor Patient Prognosis

**Figure 4:**
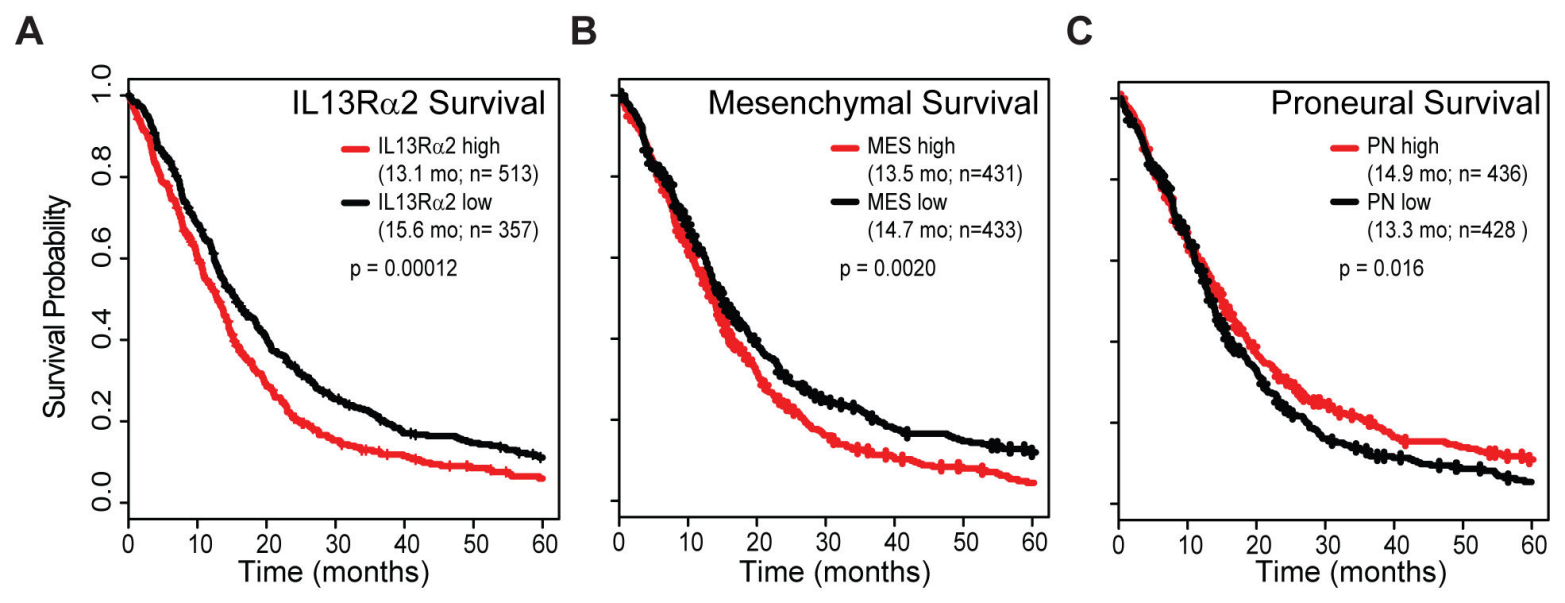
Over-expression of IL13Rα2 is associated with decreased patient survival. (A) Kaplan-Meier survival plot for patients with GBM segregated based on ‘high’ versus ‘low’ IL13Rα2 expression as determined using non-linear least-squares regression (Table S1 in File S1). Kaplan-Meier plots for the same patient cohort evaluated in panel (A) are segregated based on (B) mesenchymal signature gene expression or (C) proneural signature gene expression, with ’high‘ and ’low‘ expression determined by median expression level.

To evaluate whether IL13Rα2 expression impacts patient prognosis, expression array data from eight studies of WHO Grade IV GBMs [6,19-25] (Table 1) was aggregated and stratified based upon frequencies of over-expression determined using our NLS model (Figure 1C). We observed that IL13Rα2 over-expression is negatively correlated with patient outcome, decreasing median patient survival time by 2.5 months (Figure 4A, p=0.00012), with a 13.1 month median survival time for ‘high’ IL13Rα2 expression (CI 95%: 12.1-14.5 mo, n=513) as compared to 15.6 months for ‘low’ IL13Rα2 expression (CI 95%: 13.6 - 18.6 mo, n=357). Additionally, the ‘high’ expression group shows a 34.3% increase (CI 95%: 15.6%-56.2%) in hazard risk as compared to the ‘low’ group. Further, when the cohort was expanded to include both WHO III and IV high-grade tumors, ‘high’ IL13Rα2 expression is associated with a larger (5.8 month) decrease in median survival time (p=6.8 x 10^-10^) (Figure S8A in File S1). The IL13Rα2 survival difference in GBMs (WHO IV) is very consistent across multiple cohorts. This is reflected in IL13Rα2 expression having a survival impact that is within the top ~1% of total expression array probes in the combined eight-cohort GBM dataset when these are ranked by statistical significance of survival association (ranking 236 of 22,215 gene probes; Dataset S7). These findings demonstrate for the first time that IL13Rα2 expression in high-grade gliomas is a prognostic indicator of poor patient survival, and are in agreement with a recent finding that high IL13Rα2 expression is also associated with poor patient prognosis in colorectal cancer [38]. 

We next assessed whether the decrease in patient survival associated with IL13Rα2 expression relates to its correlation with mesenchymal signature gene expression. Verhaak et al. [5] reported no significant survival differences between the TCGA subtypes, and our 4-way survival analysis of the TCGA training dataset was consistent with this conclusion (p=0.255, Figure S8B in File S1). We noted, however, that the survival difference between mesenchymal and proneural tumors in the (relatively small) training dataset is nearly significant (p=0.0514, Figure S8B in File S1), suggesting that statistical significance could be reached with a larger patient cohort. We therefore examined patient survival in the 870 WHO Grade IV tumor cohort [6,19-25] previously evaluated for the IL13Rα2 survival analysis above. When these data were stratified based on means of averaged mesenchymal (Figure 4B) and proneural (Figure 4C) signature gene expression, ‘high’ mesenchymal expression was associated with 1.2 mo survival decrease (p=0.0020, HR=1.26) and ‘high’ proneural expression with a 1.6 mo survival increase (p=0.016, HR=0.83). Thus, high expression of mesenchymal signature genes is associated with poor prognosis and, conversely, high proneural signature gene expression is associated with better prognosis. Significantly, IL13Rα2 single gene expression is associated with a stronger survival differential than either the averaged mesenchymal or proneural proneural signature gene sets.

## Discussion

Focusing on IL13Rα2, a clinically relevant glioma target being pursued for therapeutic development [11,28,39-41], we have taken a bioinformatics approach to better understand the intrinsic biology of tumors with high IL13Rα2 expression, and to gain insight into how this may affect inter-patient IL13Rα2 heterogeneity in gliomas. We find that within GBM, IL13Rα2 is over-expressed in approximately 58% of gliomas (Figure 1C, Table S1 in File S1), and displays a distinct bimodal expression pattern (Figure 1A) [8]. Further, IL13Rα2 expression increases with glioma malignancy grade (Figure 1) [34] and is associated with decreased long-term survival, a single gene correlation stronger than seen for the mesenchymal signature genes (Figure 4). IL13Rα2 ranks at ~1% level of total gene probes with regard to patient survival (Dataset S7), thus establishing IL13Rα2 as a prognostic indicator for poor patient outcome. 

Our data also indicate that inter-patient heterogeneity of IL13Rα2 expression should be viewed in the context of molecular subtypes of high-grade gliomas [42]. By several measures, IL13Rα2 expression is most closely correlated, but not limited to, expression of mesenchymal signature genes [5,6] as determined by principal component analyses (Figure 2A, B, and Table S2 in File S1), correlation analysis with subtype signature genes (Figure 2C,D, Table S3 in File S1), and a cohort of individual patients’ tumors (Figure 2E, 2F). Further, for a panel of patient-derived IL13Rα2-positive and -negative cell lines, its expression is closely correlated with mesenchymal signature gene expression, suggesting that the association with the mesenchymal subclass can be intrinsic to tumor cells themselves (Figure 3).  IPA enrichment analysis also indicates that pathways defined by genes associated with IL13Rα2 over-expression in patient samples (Table S5 in File S1) and IL13Rα2-positive cell lines (Table S6 in File S1) are shared with mesenchymal (Table S7 in File S1), but not proneural tumor subclasses (Table S9 in File S1), and further that these shared pathways are often related to immune system functions (Tables S4-S6 in File S1).

 The Verhaak et al. [5] and Phillips et al. [6] signature gene sets were defined based on different underlying algorithms: unsupervised clustering for the former and patient prognosis for the later. We therefore find it interesting that the correlations of IL13Rα2 expression with mesenchymal and proneural signature genes for both schemes is anti-phasic: strongly associated with the mesenchymal subclass and excluded from the proneural (Figure 2C, 2D). The mesenchymal and proneural subclasses are the two most molecularly distinct, and this pattern of IL13Rα2 expression is almost certainly a reflection of strong differences in the intrinsic biology of these tumors. At the same time, IL13Rα2 associations with the intervening subclasses differed [42]. For the Verhaak et al. [5] analysis, segmenting tumors by unsupervised clustering yielded two additional tumor classes, classical and neural, across which IL13Rα2 association was a continuum of increasing correlation from proneural to mesenchymal (Figure 2E). Phillips et al. [6], however, defined their classes based on prognosis followed by cluster analysis, with proliferation (proliferative) and angiogenesis (mesenchymal) associating with poor patient outcome. In this more physiological tumor segmentation, IL13Rα2 expression was well correlated with both the mesenchymal and proliferative signature genes, consistent with our observations linking IL13Rα2 expression to poor patient prognosis. Unfortunately, in this bioinformatics study we could not further explore (for example, by IPA analysis) the IL13Rα2 related properties of the proliferative phenotype as defined by Phillips et al. [6], because of the limited number of gene available for further analyses. Thus we conclude that while IL13Rα2 expression is not restricted to mesenchymal subclass tumors, its expression closely correlates with mesenchymal signature gene expression and thus presumably mesenchymal properties of a tumor.

Mesenchymal signature gene expression is known to be associated with immune-related inflammatory responses, dissemination, angiogenesis, and poor prognosis [5,6,43].  Significantly, we find that pathways shared by IL13Rα2 over-expressing cell lines and tumor samples, and those associated with mesenchymal signature genes, all show strong enrichment for immunological processes and inflammation (Tables 2 and 3; Tables S5, S6 and S7 in File S1).  Strong association with the Hepatic Fibrosis IPA pathway is a finding consistent with previous studies of IL13Rα2 biology showing it, despite its assigned role as a decoy receptor, to be associated with fibrosis [44]. Another consistent association is with genes linked to TREM1 signaling, a process important in initiation of inflammatory immune responses [45], and also up-regulated in glioblastoma tissue [46].  These observations suggest that enhanced immune activation and inflammatory responses may underlie the positive association between IL13Rα2 and the mesenchymal subtype. 

Our results indicate that IL13Rα2 is preferentially expressed at high levels in a subset of patient tumors of high virulence in the context of mesenchymal-associated genes. Given the relationship of IL13Rα2 to immune-related pathways indicated by IPA analysis, the involvement of IL13Rα2 expression in Th2 inflammatory diseases [13,47], and the suggested role of inflammation in epithelial-to-mesenchymal transition and cancer progression [48,49], it is tempting to suggest that IL13Rα2 expression may mark tumor progression [17,38] as a reflection of activation of inflammation-related signaling pathways. This work therefore provides a framework within which future studies can further investigate our hypothesized association of IL13Rα2 expression and immune pathway activation.

Immunotherapy strategies targeting IL13Rα2 for brain tumors are being clinically pursued [11,28,39-41] based on its selective expression on malignant versus normal brain tissue [7-10]. Our findings now suggest that IL13Rα2 targeting will be skewed towards (but not limited to) the mesenchymal glioma subtype. Our group has developed a platform to target high-grade gliomas based on IL13Rα2 expression by genetically modifying T cells to express an IL13Rα2-specific chimeric antigen receptor (CAR), IL13-zetakine [11,28]. These engineered IL13-zetakine^+^ T cells exhibit potent MHC-independent, IL13Rα2-specific cytolytic activity against both stem-like and differentiated glioma cells, and induce regression of established glioma xenografts *in vivo* [11,28]. Observations from this current work lead us to infer that IL13-zetakine CTLs can efficiently target and kill IL13Rα2^pos^ glioblastoma cells expressing mesenchymal gene signature phenotypes. The IL13Rα2^pos^ and mesenchymal tumor lines PBT015, PBT017-4, PBT030 and U251T (Figure 3) were shown in our previous work to be lysed by IL13-zetakine^+^ T cells, and co-injected IL13-zetakine T cells ablate *in vivo* tumor initiation of these same lines [11]. Moreover in mice, adoptive transfer of IL13-zetakine T cells display potent anti-tumor activity against established IL13Rα2^pos^ PBT017-4 and PBT030-2 xenografts which express a mesenchymal gene expression profile [11]. Future work will be directed towards evaluating clinical responses in patients treated in our first-in-human pilot clinical trials [41] with respect to glioma molecular subtypes and tumor heterogeneity. 

By describing the molecular subset of GBM over-expressing IL13Rα2 and the intrinsic biology of these tumors, the studies presented here provide a greater understanding of the potential therapeutic utility of IL13Rα2-targeting therapies for this patient population, and provide the foundation for future refinements of these therapies.

## Supporting Information

File S1 **Tables S1-S9 and Figures S1-S8.** Table S1, Frequency of IL13Rα2 over-expression for GBM based on NLS Model. Table S2, Average Distances between IL13Rα2 and Signature Genes. Table S3, Numbers of glioma Subtype Signature Genes that Correlate with IL13Rα2 Expression. Table S4, Numbers of Canonical Pathways Associated with Mesenchymal, Classical and Proneural Signature Genes in Common with IL13Rα2 Over-expression. Table S5, Canonical Pathways Associated with Genes Positively Correlated with IL13Rα2 in Patient Cohorts. Table S6, Canonical Pathways Associated with Genes Up-regulated in IL13Rα2-positive Cell Lines. Table S7, Canonical Pathways Associated with Mesenchymal Signature Genes and their Relation to those Associated with Immune Activation and IL13Rα2 Expression. Table S8, Canonical Pathways associated with Classical Signature Genes in Relation to those associated with Immune Activation and IL13Rα2 Expression. Table S9, Canonical Associated with Proneural Signature Genes and their Relation to those Associated with Immune Activation and IL13Rα2 Expression. Figure S1, Expression of IL13Rα2 does not change with tumor recurrence. Figure S2, Validation of Silhouette plot analysis correlating expression of an individual gene with glioma subtypes. Figure S3, Correlation of IL13Rα2 versus average mesenchymal signature gene expression for individual study cohorts. Figure S4, Dot plots showing distributions of RMA-normalized IL13Rα2 expression levels across molecularly-defined subtypes. Figure S5, Density of correlation coefficients for average mesenchymal signature gene expression per study. Figure S6, Comparison of a DLL3, EGFR, and CHI3L1 gene expression with respect to glioma subclass and malignancy grade. Figure S7, Pathways identified by IPA for mesenchymal signature genes are also highly enriched for genes correlated with IL13Rα2 over-expression. Figure S8, glioma survival analyses.
(PDF)

File S2 **Datasets S1-S7.** Dataset S1, Probes for TCGA signature genes correlated with IL13Rα2. Dataset S2, Probes for Philips signature genes correlated with IL13Rα2. Dataset S3, Gene Signature Probes used for TCGA Subtypes. Dataset S4, Gene Signature Probes used for Phillips Subtypes. Dataset S5, Dataset S5: Number of Significant Correlations per Gene in Eight Study Cohorts. Dataset S6, Differential gene expression statistics for IL13Rα2-positive versus ‑negative cell lines. Dataset S7, Genome-wide survival analysis.
(XLSX)

## References

[1] Natsume A, Kinjo S, Yuki K, Kato T, Ohno M et al. (2011) Glioma-initiating cells and molecular pathology: implications for therapy. Brain Tumor Pathology 28: 1-12. doi:10.1007/s10014-010-0011-3. PubMed: 21274750. 21274750

[2] McLendon RE, Rich JN (2011) Glioblastoma Stem Cells: A Neuropathologist&#39 s View. Journal of Oncology 2011 10.1155/2011/397195 PMC2971570 21052560

[3] Van Meir EG, Hadjipanayis CG, Norden AD, Shu H-K, Wen PY et al. (2010) Exciting New Advances in Neuro-Oncology: The Avenue to a Cure for Malignant Glioma. CA Cancer J Clin 60: 166-193. doi:10.3322/caac.20069. PubMed: 20445000. 20445000 PMC2888474

[4] Hodgson JG, Yeh R-F, Ray A, Wang NJ, Smirnov I et al. (2009) Comparative analyses of gene copy number and mRNA expression in glioblastoma multiforme tumors and xenografts. Neuro-Oncology 11: 477-487. doi:10.1215/15228517-2008-113. PubMed: 19139420. 19139420 PMC2765338

[5] Verhaak RGW, Hoadley KA, Purdom E, Wang V, Qi Y et al. (2010) Integrated Genomic Analysis Identifies Clinically Relevant Subtypes of Glioblastoma Characterized by Abnormalities in PDGFRA, IDH1, EGFR, and NF1. Cancer Cell 17: 98-110. doi:10.1016/j.ccr.2009.12.020. PubMed: 20129251. 20129251 PMC2818769

[6] Phillips HS, Kharbanda S, Chen R, Forrest WF, Soriano RH et al. (2006) Molecular subclasses of high-grade glioma predict prognosis, delineate a pattern of disease progression, and resemble stages in neurogenesis. Cancer Cell 9: 157 - 173. doi:10.1016/j.ccr.2006.02.019. PubMed: 16530701. 16530701

[7] Debinski W, Gibo DM, Hulet SW, Connor JR, Gillespie GY (1999) Receptor for Interleukin 13 Is a Marker and Therapeutic Target for Human High-Grade Gliomas. Clin Cancer Res 5: 985-990. PubMed: 10353730. 10353730

[8] Jarboe JS, Johnson KR, Choi Y, Lonser RR, Park JK (2007) Expression of Interleukin-13 Receptor a2 in Glioblastoma Multiforme: Implications for Targeted Therapies. Cancer Res 67: 7983-7986. doi:10.1158/0008-5472.CAN-07-1493. PubMed: 17804706. 17804706

[9] Joshi BH, Puri RA, Leland P, Varricchio F, Gupta G et al. (2008) Identification of interleukin-13 receptor α2 chain overexpression in situ in high-grade diffusely infiltrative pediatric brainstem glioma. Neuro-Oncology 10: 265-274. doi:10.1215/15228517-2007-066. PubMed: 18430795. 18430795 PMC2563049

[10] Kawakami M, Kawakami K, Takahashi S, Abe M, Puri RK (2004) Analysis of interleukin-13 receptor α2 expression in human pediatric brain tumors. Cancer 101: 1036-1042. doi:10.1002/cncr.20470. PubMed: 15329913. 15329913

[11] Brown CE, Starr R, Aguilar B, Shami AF, Martinez C et al. (2012) Stem-like tumor-initiating cells isolated from IL13Ralpha2 expressing gliomas are targeted and killed by IL13-zetakine-redirected T Cells. Clin Cancer Res 18: 2199-2209. doi:10.1158/1078-0432.CCR-11-1669. PubMed: 22407828. 22407828 PMC3578382

[12] Lupardus PJ, Birnbaum ME, Garcia KC (2010) Molecular basis for shared cytokine recognition revealed in the structure of an unusually high affinity complex between IL-13 and IL-13Ralpha2. Structure 18: 332-342. doi:10.1016/j.str.2010.01.003. PubMed: 20223216. 20223216 PMC2850121

[13] Tabata Y, Khurana Hershey GK (2007) IL-13 receptor isoforms: Breaking through the complexity. Curr Allergy Asthma Rep 7: 338-345. doi:10.1007/s11882-007-0051-x. PubMed: 17697639. 17697639

[14] Chiaramonte MG, Mentink-Kane M, Jacobson BA, Cheever AW, Whitters MJ et al. (2003) Regulation and Function of the Interleukin 13 Receptor α 2 During a T Helper Cell Type 2-dominant Immune Response. J Exp Med 197: 687-701. doi:10.1084/jem.20020903. PubMed: 12642601. 12642601 PMC2193852

[15] Fichtner-Feigl S, Strober W, Kawakami K, Puri RK, Kitani A (2006) IL-13 signaling through the IL-13[alpha]2 receptor is involved in induction of TGF-[beta]1 production and fibrosis. Nat Med 12: 99-106. doi:10.1038/nm1332. PubMed: 16327802. 16327802

[16] Fichtner-Feigl S, Terabe M, Kitani A, Young CA, Fuss I et al. (2008) Restoration of Tumor Immunosurveillance via Targeting of Interleukin-13 Receptor-α2. Cancer Res 68: 3467-3475. doi:10.1158/0008-5472.CAN-07-5301. PubMed: 18451175. 18451175 PMC2746996

[17] Fujisawa T, Joshi BH, Puri RK (2011) IL-13 regulates cancer invasion and metastasis through IL-13Rα2 via ERK/AP-1 pathway in mouse model of human ovarian cancer. Int J Cancer: n/a-n/a 10.1002/ijc.26366 21858811

[18] Hsi LC, Kundu S, Palomo J, Xu B, Ficco R et al. (2011) Silencing IL-13Ralpha2 promotes glioblastoma cell death via endogenous signaling. Mol Cancer Ther 10: 1149-1160. doi:10.1158/1535-7163.MCT-10-1064. PubMed: 21596889. 21596889 PMC3132296

[19] Freije WA, Castro-Vargas FE, Fang Z, Horvath S, Cloughesy T et al. (2004) Gene expression profiling of gliomas strongly predicts survival. Cancer Res 64: 6503 - 6510. doi:10.1158/0008-5472.CAN-04-0452. PubMed: 15374961. 15374961

[20] Gravendeel LAM, Kouwenhoven MCM, Gevaert O, de Rooi JJ, Stubbs AP et al. (2009) Intrinsic Gene Expression Profiles of Gliomas Are a Better Predictor of Survival than Histology. Cancer Res 69: 9065-9072. doi:10.1158/0008-5472.CAN-09-2307. PubMed: 19920198. 19920198

[21] Lee Y, Scheck AC, Cloughesy TF, Lai A, Dong J et al. (2008) Gene expression analysis of glioblastomas identifies the major molecular basis for the prognostic benefit of younger age. BMC Med Genomics 1: 52. doi:10.1186/1755-8794-1-52. PubMed: 18940004. 18940004 PMC2596165

[22] Murat A, Migliavacca E, Gorlia T, Lambiv WL, Shay T et al. (2008) Stem Cell-Related "Self-Renewal" Signature and High Epidermal Growth Factor Receptor Expression Associated With Resistance to Concomitant Chemoradiotherapy in Glioblastoma. J Clin Oncol 26: 3015-3024. doi:10.1200/JCO.2007.15.7164. PubMed: 18565887. 18565887

[23] Petalidis LP, Oulas A, Backlund M, Wayland MT, Liu L et al. (2008) Improved grading and survival prediction of human astrocytic brain tumors by artificial neural network analysis of gene expression microarray data. Mol Cancer Ther 7: 1013-1024. doi:10.1158/1535-7163.MCT-07-0177. PubMed: 18445660. 18445660 PMC2819720

[24] Sun L, Hui A-M, Su Q, Vortmeyer A, Kotliarov Y et al. (2006) Neuronal and glioma-derived stem cell factor induces angiogenesis within the brain. Cancer Cell 9: 287-300. doi:10.1016/j.ccr.2006.03.003. PubMed: 16616334. 16616334

[25] The Cancer Genome Atlas Research Network (2008) Comprehensive genomic characterization defines human glioblastoma genes and core pathways. Nature 455: 1061-1068. doi:10.1038/nature07385. PubMed: 18772890. 18772890 PMC2671642

[26] Brown CE, Starr R, Martinez C, Aguilar B, D'Apuzzo M et al. (2009) Recognition and Killing of Brain Tumor Stem-Like Initiating Cells by CD8+ Cytolytic T Cells. Cancer Res 69: 8886-8893. doi:10.1158/0008-5472.CAN-09-2687. PubMed: 19903840. 19903840 PMC2789196

[27] Hemmati HD, Nakano I, Lazareff JA, Masterman-Smith M, Geschwind DH et al. (2003) Cancerous stem cells can arise from pediatric brain tumors. Proc Natl Acad Sci USA 100: 15178-15183. doi:10.1073/pnas.2036535100. PubMed: 14645703. 14645703 PMC299944

[28] Kahlon KS, Brown C, Cooper LJN, Raubitschek A, Forman SJ et al. (2004) Specific Recognition and Killing of Glioblastoma Multiforme by Interleukin 13-Zetakine Redirected Cytolytic T Cells. Cancer Res 64: 9160-9166. doi:10.1158/0008-5472.CAN-04-0454. PubMed: 15604287. 15604287

[29] Stastny MJ, Brown CE, Ruel C, Jensen MC (2007) Medulloblastomas Expressing IL13R[alpha]2 are Targets for IL13-zetakine+ Cytolytic T Cells. J Pediatr Hematol/Oncol 29: 610-677 1097/MPH.1090b1013e3181468c3181468 doi:10.1097/MPH.0b013e3181468c68. 17921847

[30] Deng X, Warden C, Liu Z, Zhang I, Bravo Yuan Y-C: biomarkers recognition and validation online (in preparation).

[31] Irizarry RA, Hobbs B, Collin F, Beazerâ€Barclay YD, Antonellis KJ, et al (2003) Exploration, normalization, and summaries of high density oligonucleotide array probe level data. Biostatistics 4: 249-264. doi:10.1093/biostatistics/4.2.249. PubMed: 12925520. 12925520

[32] R Development Core Team (2013) R: A Language and Environment for Statistical Computing. Vienna, Austria: R Foundation for Statistical Computing.

[33] Benjamini Y, Hochberg Y (1995) Controlling the False Discovery Rate: A Practical and Powerful Approach to Multiple Testing. J R Stat Soc B Stat Methodol) 57: 289-300.

[34] Wykosky J, Gibo DM, Stanton C, Debinski W (2008) Interleukin-13 Receptor α2, EphA2, and Fos-Related Antigen 1 as Molecular Denominators of High-Grade Astrocytomas and Specific Targets for Combinatorial Therapy. Clin Cancer Res 14: 199-208. doi:10.1158/1078-0432.CCR-07-1990. PubMed: 18172271. 18172271

[35] Beier D, Hau P, Proescholdt M, Lohmeier A, Wischhusen J et al. (2007) CD133+ and CD133− Glioblastoma-Derived Cancer Stem Cells Show Differential Growth Characteristics and Molecular Profiles. Cancer Res 67: 4010-4015. doi:10.1158/0008-5472.CAN-06-4180. PubMed: 17483311. 17483311

[36] Lottaz C, Beier D, Meyer K, Kumar P, Hermann A et al. (2010) Transcriptional Profiles of CD133+ and CD133− Glioblastoma-Derived Cancer Stem Cell Lines Suggest Different Cells of Origin. Cancer Res 70: 2030-2040. doi:10.1158/0008-5472.CAN-09-1707. PubMed: 20145155. 20145155

[37] Beier CP, Kumar P, Meyer K, Leukel P, Bruttel V et al. (2012) The Cancer Stem Cell Subtype Determines Immune Infiltration of Glioblastoma. Stem Cells Dev, 21: 2753–61. PubMed: 22676416. 22676416 10.1089/scd.2011.0660 PMC3464079

[38] Barderas R, Bartolome RA, Fernandez-Acenero MJ, Torres S, Casal I (2012) High expression of IL-13 receptor α2 in colorectal cancer is associated with invasion, liver metastasis and poor prognosis. Cancer Res. 10.1158/0008-5472.CAN-11-4090 22505647

[39] Kunwar S, Chang S, Westphal M, Vogelbaum M, Sampson J et al. (2010) Phase III randomized trial of CED of IL13-PE38QQR vs Gliadel wafers for recurrent glioblastoma. Neuro Oncol 12: 871-881. doi:10.1093/neuonc/nop054. PubMed: 20511192. 20511192 PMC2940677

[40] Saka M, Amano T, Kajiwara K, Yoshikawa K, Ideguchi M et al. (2009) Vaccine therapy with dendritic cells transfected with Il13ra2 mRNA for glioma in mice. J Neurosurg 113: 270-279. PubMed: 19895199. 10.3171/2009.9.JNS09708 19895199

[41] Yaghoubi SS, Jensen MC, Satyamurthy N, Budhiraja S, Paik D et al. (2009) Noninvasive detection of therapeutic cytolytic T cells with 18F-FHBG PET in a patient with glioma. Nat Clin Pract Oncol 6: 53-58. doi:10.1038/ncponc1278. PubMed: 19015650. 19015650 PMC3526373

[42] Huse JT, Phillips HS, Brennan CW (2011) Molecular subclassification of diffuse gliomas: seeing order in the chaos. Glia 59: 1190-1199. doi:10.1002/glia.21165. PubMed: 21446051. 21446051

[43] Colman H, Zhang L, Sulman EP, McDonald JM, Shooshtari NL et al. (2010) A multigene predictor of outcome in glioblastoma. Neuro Oncol 12: 49-57. doi:10.1093/neuonc/nop007. PubMed: 20150367. 20150367 PMC2940562

[44] MacDonald TT (2006) Decoy receptor springs to life and eases fibrosis. Nat Med 12: 13-14. doi:10.1038/nm0106-13. PubMed: 16397542. 16397542

[45] Ford JW, McVicar DW (2009) TREM and TREM-like receptors in inflammation and disease. Curr Opin Immunol 21: 38-46. doi:10.1016/j.coi.2009.01.009. PubMed: 19230638. 19230638 PMC2723941

[46] Murat A, Migliavacca E, Hussain SF, Heimberger AB, Desbaillets I et al. (2009) Modulation of angiogenic and inflammatory response in glioblastoma by hypoxia. PLOS ONE 4: e5947. doi:10.1371/journal.pone.0005947. PubMed: 19536297. 19536297 PMC2694268

[47] Joshi BH, Hogaboam C, Dover P, Husain SR, Puri RK (2006) Role of interleukin-13 in cancer, pulmonary fibrosis, and other T(H)2-type diseases. Vitam Horm 74: 479-504. doi:10.1016/S0083-6729(06)74019-5. PubMed: 17027527. 17027527

[48] Grivennikov SI, Greten FR, Karin M (2010) Immunity, inflammation, and cancer. Cell 140: 883-899. doi:10.1016/j.cell.2010.01.025. PubMed: 20303878. 20303878 PMC2866629

[49] Lopez-Novoa JM, Nieto MA (2009) Inflammation and EMT: an alliance towards organ fibrosis and cancer progression. EMBO. Mol Med 1: 303-314. 10.1002/emmm.200900043 PMC3378143 20049734

