# Correction: Glioma IL13Rα2 Is Associated with Mesenchymal Signature Gene Expression and Poor Patient Prognosis

**DOI:** 10.1371/journal.pone.0204463

**Published:** 2018-09-17

**Authors:** The PLOS ONE Staff

Table 2, “IPA Pathways Associated With IL13Rα2 Expression in Patient Tumor Samples, IL13Rα2-positive Cell Lines, and Mesenchymal Signature Genes,” incorrectly appears as a duplicate of Table 1. The figure caption appears correctly. Please see the corrected Table 2 here.

**Table 2:** IPA Pathways Associated with IL13Rα2 Expression in Patient Tumor Samples, IL13Rα2-positive Cell Lines, and Mesenchymal Signature Genes.

Rank	IL13Rα2-positive Cell Lines*^,^^1^	IL13Rα2 Over-expressing Patient Tumors*^,^^2^	Mesenchymal Signature Genes*^,^^3^
1	NRF2-mediated Oxidative Stress Response	**Antigen Presentation Pathway**	**Hepatic Fibrosis / Hepatic Stellate Cell Activation**
2	**Hepatic Fibrosis / Hepatic Stellate Cell Activation**	Cell Cycle Control of Chromosomal Replication	**TREM1 Signaling**
3	Caveolar-mediated Endocytosis Signaling	**Cytotoxic T Lymphocyte-mediated Apoptosis of Target Cells**	**Dendritic Cell Maturation**
4	**Antigen Presentation Pathway**	Protein Ubiquitination Pathway	**IL-6 Signaling**
5	p38 MAPK Signaling	Cdc42 Signaling	HMGB1 Signaling
6	**Communication between Innate and Adaptive Immune Cells**	**Hepatic Fibrosis / Hepatic Stellate Cell Activation**	**IL-10 Signaling**
7	Death Receptor Signaling	**TREM1 Signaling**	Coagulation System
8	**TREM1 Signaling**	Atherosclerosis Signaling	NF-κB Signaling

*FDR < 0.05; Immune function-related pathways are indicated in bold and shading.

^1^IPA pathways associated with differential gene expression between IL13Rα2-positive and IL13Rα2-negative cell lines (|fold-change| > 1.5; FDR <0.05).

^2^IPA pathways associated with differential gene expression for patient samples, defined as genes positively correlated with IL13Rα2 in at least 4 independent cohorts.

^3^IPA pathways associated with canonical mesenchymal signature genes defined by Verhaak et al. [5]. Note that Hepatic Fibrosis and TREM1 signaling are among the top 8 enriched pathways for all 3 gene lists.
