# Supplementary material for: Glioma IL13Rα2 Is Associated with Mesenchymal Signature Gene Expression and Poor Patient Prognosis
Source: PLoS One. 2013 Oct 18;8(10):e77769. doi: 10.1371/journal.pone.0077769 (PMC3800130; doi:10.1371/journal.pone.0077769)
Supplement: File S1 — Tables S1-S9 and Figures S1-S8. Table S1, Frequency of IL13Rα2 over-expression for GBM based on NLS Model. Table S2, Average Distances between IL13Rα2 and Signature Genes. Table S3, Numbers of glioma Subtype Signature Genes that Correlate with IL13Rα2 Expression. Table S4, Numbers of Canonical Pathways Associated with Mesenchymal, Classical and Proneural Signature Genes in Common with IL13Rα2 Over-expression. Table S5, Canonical Pathways Associated with Genes Positively Correlated with IL13Rα2 in Patient Cohorts. Table S6, Canonical Pathways Associated with Genes Up-regulated in IL13Rα2-positive Cell Lines. Table S7, Canonical Pathways Associated with Mesenchymal Signature Genes and their Relation to those Associated with Immune Activation and IL13Rα2 Expression. Table S8, Canonical Pathways associated with Classical Signature Genes in Relation to those associated with Immune Activation and IL13Rα2 Expression. Table S9, Canonical Associated with Proneural Signature Genes and their Relation to those Associated with Immune Activation and IL13Rα2 Expression. Figure S1, Expression of IL13Rα2 does not change with tumor recurrence. Figure S2, Validation of Silhouette plot analysis correlating expression of an individual gene with glioma subtypes. Figure S3, Correlation of IL13Rα2 versus average mesenchymal signature gene expression for individual study cohorts. Figure S4, Dot plots showing distributions of RMA-normalized IL13Rα2 expression levels across molecularly-defined subtypes. Figure S5, Density of correlation coefficients for average mesenchymal signature gene expression per study. Figure S6, Comparison of a DLL3, EGFR, and CHI3L1 gene expression with respect to glioma subclass and malignancy grade. Figure S7, Pathways identified by IPA for mesenchymal signature genes are also highly enriched for genes correlated with IL13Rα2 over-expression. Figure S8, glioma survival analyses. (PDF) [file pone.0077769.s001.pdf]

Table S1: Frequency of IL13Rα2 over-expression for GBM based on NLS Model.

Study	Tumor (n)	m_{over}	m_{under}	S	Frequency
Freije	50	11.99	8.15	1.54	55%
Gravendeel	159	9.98	5.37	1.39	64%
Lee HG-U133A	191	8.41	5.11	0.98	37%
Murat	70	9.45	6.11	1.09	53%
Petalidis*	35	7.39	4.68	S1 = 1.06;<br>S2 = 0.75	74%
Phillips	56	9.81	4.98	1.84	73%
Sun	59	10.55	7.08	1.4	54%
TCGA HG-U133A	339	9.05	4.75	1.3	65%

$\mathbf{m_{over}}$ = mean expression of IL13Rα2 in the population of patients showing over-expression of IL13Rα2

$\mathbf{m_{under}}$ = mean expression of IL13Rα2 in the population of patients showing under-expression of IL13Rα2

S = standard deviation for each population of patients, assumed to be the same for both over- and under-expressing populations (*except for Petalidis where S1 and S2 are for over- and under-expressing patients, respectively).

Table S2: Average Distances between IL13Rα2 and Signature Genes

	Euclidian Distance	Pearson Dissimilarity Distance
**Verhaak TCGA**		
Proneural	51.3	0.91
Neural	49.1	0.71
Classical	45.2	0.37
Mesenchymal	**41.5**	**0.28**
**Phillips**		
Proneural	68.9	0.74
Proliferative	44.2	0.31
Mesenchymal	**43.2**	**0.26**

Distances are calculated for averaged signature gene expression as defined by Verhaak et al. [1] and Phillips et al. [2] in a dataset combining those of Gravendeel et al. [3] and Sun [4]..

Table S3: Numbers of Glioma Subtype Signature Genes that Correlate with IL13Rα2 Expression.

	Total Genes per subtype*	Positive Correlation§	Negative Correlation§	Mixed Correlation§	No Correlation§
**Verhaak TCGA**					
Proneural	178	5 (2.8%)	132 (74.2%)	33 (18.5%)	8 (4.5%)
Neural	128	28 (21.9%)	45 (35.2%)	55 (42.97%)	0 (0%)
Classical	162	51 (31.5%)	53 (32.7%)	51 (31.5%)	7 (4.3%)
Mesenchymal	215	171 (79.5%)	3 (1.4%)	40 (18.6%)	1 (0.5%)
**Phillips**					
Proneural	14	0 (0%)	13 (92.9%)	0 (0%)	1 (7.1%)
Proliferative	5	4 (80%)	0 (0%)	1 (20%)	0 (0%)
Mesenchymal	15	12 (80%)	0 (0%)	3 (20%)	0 (0%)

*Number of genes per subtype evaluated for correlation with IL13Rα2 expression. Total number of genes assessed was 685 encompassing 8 studies [2-9].

§The number of genes and percentage of total (shown in parenthesis) whose expression show "positive correlation" and "negative correlation" with glioma IL13Rα2 expression ($p < 0.05$) in all eight datasets. One positive or negative correlation was sufficient to define a gene as showing positive or negative association with IL13Rα2. "Mixed correlations" were defined as genes with inconsistent probe correlations between the 8 studies, that being one probe/study showed a significant positive correlation (FDR < 0.05) and another probe/study showed a negative correlation (FDR < 0.05). "No correlations" were defined as genes that showed no significant probe correlations, either positive or negative, for all 8 studies.

Table S4: Numbers of Canonical Pathways Associated with Mesenchymal, Classical and Proneural Signature Genes in Common with IL13Rα2 Over-expression[1].

	**MES[2]**	**IL13Rα2^{pos} Cell Lines[3]**	**IL13Rα2^{pos} Patient Samples[4]**	**PN[5]**	**CL[6]**
**MES[2]**	X	MES: 56;<br>**Overlap: 15**;<br>Cell Lines: 8;<br>***p = 3.75 x 10^{-8}***	MES: 54;<br>**Overlap: 17**;<br>Patient: 28;<br>***p = 0.00028***	MES: 71;<br>**Overlap: 0;**<br>PN: 7;<br>*p = 0.76*	MES: 68;<br>**Overlap: 3;**<br>CL: 11;<br>*p = 0.256*
**IL13Ra2^{pos} Cell Lines[3]**		X	Cell Lines: 9;<br>**Overlap: 14**;<br>Patients: 30;<br>***p = 1.3 x 10^{11}***	Cell Lines: 23;<br>**Overlap: 0**;<br>PN: 7;<br>*p = 0.35*	Cell Lines: 22;<br>**Overlap: 1;**<br>CL: 13;<br>*p = 0.203*
**IL13Rα2^{pos} Patient Samples[4]**			X	Patients: 45;<br>**Overlap: 0**;<br>PN: 7;<br>*p = 0.59*	Patients: 44;<br>**Overlap: 1**;<br>CL: 13;<br>*p = 0.507*
**PN[5]**				X	PN: 5;<br>**Overlap: 2;**<br>CL: 12;<br>***p = 0.00126***
**CL[6]**					X

For IPA analysis 382 canonical pathways were evaluated. Note that no significant IPA pathways (FDR < 0.05) are enriched for the neural signature gene list of Verhaak et al. [1].

[2] MES: IPA canonical pathways (total of 71 with FDR < 0.05) associated with expression of mesenchymal signature genes (n = 216).

[3] IL13Rα2^{pos} Cell Lines: IPA pathways (total of 23) associated with genes up-regulated in IL13Rα2-positive cell lines (fold-change| > 1.5 and FDR < 0.05)

[4] IL13Rα2^{pos} Patient Samples: IPA pathways (total of 45) associated with genes up-regulated in patient samples in at least four of eight cohorts (FDR < 0.05)

[5] PN : IPA canonical pathways (total of 7 with FDR < 0.05) associated with expression of proneural signature genes (n = 178)

[6] CL: IPA canonical pathways (total of 14 with FDR < 0.05) associated with expression of classical signature genes (n = 162)

Table S5: Canonical Pathways Associated with Genes Positively Correlated with IL13Rα2 in Patient Cohorts (FDR < 0.05). Immune-related pathways are shown in **bold red**, and pathways common to IL13Rα2 expression in patient cohorts and IL13Rα2-positive cell lines are highlighted.

	Ingenuity Canonical Pathways	FDR
**1**	**Antigen Presentation Pathway**	**3.98E-05**
2	Cell Cycle Control of Chromosomal Replication	3.98E-05
**3**	**Cytotoxic T Lymphocyte-mediated Apoptosis of Target Cells**	**0.00012**
4	Protein Ubiquitination Pathway	0.000646
5	Cdc42 Signaling	0.00123
**6**	**Hepatic Fibrosis / Hepatic Stellate Cell Activation**	**0.00123**
**7**	**TREM1 Signaling**	**0.002138**
8	Atherosclerosis Signaling	0.002455
9	Pyrimidine Metabolism	0.002512
10	Role of Pattern Recognition Receptors in Recognition of Bacteria and Viruses	0.003467
**11**	**Type I Diabetes Mellitus Signaling**	**0.003631**
12	Role of CHK Proteins in Cell Cycle Checkpoint Control	0.003802
13	HMGB1 Signaling	0.008511
14	Cell Cycle: G2/M DNA Damage Checkpoint Regulation	0.010715
**15**	**Crosstalk between Dendritic Cells and Natural Killer Cells**	**0.010965**
16	Actin Nucleation by ARP-WASP Complex	0.010965
**17**	**Graft-versus-Host Disease Signaling**	**0.010965**
18	Inhibition of Angiogenesis by TSP1	0.012023
19	Hereditary Breast Cancer Signaling	0.012303
20	Mitotic Roles of Polo-Like Kinase	0.012303
21	Death Receptor Signaling	0.012303
**22**	**Acute Phase Response Signaling**	**0.012303**
23	Caveolar-mediated Endocytosis Signaling	0.012303
24	N-Glycan Biosynthesis	0.012303
25	LXR/RXR Activation	0.014125
26	Integrin Signaling	0.014125
**27**	**Tumoricidal Function of Hepatic Natural Killer Cells**	**0.014125**
**28**	**OX40 Signaling Pathway**	**0.014454**
**29**	**Allograft Rejection Signaling**	**0.015136**
**30**	**Role of Macrophages, Fibroblasts and Endothelial Cells in Rheumatoid Arthritis**	**0.018621**
**31**	**Dendritic Cell Maturation**	**0.020417**
**32**	**Complement System**	**0.022909**
33	Mismatch Repair in Eukaryotes	0.025119
**34**	**TWEAK Signaling**	**0.026915**
35	Oxidative Phosphorylation	0.027542
36	Agrin Interactions at Neuromuscular Junction	0.030903

**37**	**TNFR1 Signaling**	**0.032359**
**38**	**Communication between Innate and Adaptive Immune Cells**	**0.032359**
39	NRF2-mediated Oxidative Stress Response	0.038019
**40**	**Autoimmune Thyroid Disease Signaling**	**0.039811**
**41**	**Toll-like Receptor Signaling**	**0.040738**
**42**	**Role of IL-17A in Psoriasis**	**0.040738**
43	Role of BRCA1 in DNA Damage Response	0.040738
44	Germ Cell-Sertoli Cell Junction Signaling	0.041687
45	Mitochondrial Dysfunction	0.041687

Table S6: Canonical Pathways Associated with Genes Up-regulated in IL13Rα2-positive Cell Lines (FDR < 0.05). Immune-related pathways are shown in **bold red**, and pathways common to IL13Rα2 expression in patient cohorts and IL13Rα2-positive cell lines are highlighted.

	Ingenuity Canonical Pathways	FDR
1	NRF2-mediated Oxidative Stress Response	0.000214
**2**	**Hepatic Fibrosis / Hepatic Stellate Cell Activation**	**0.000214**
3	Caveolar-mediated Endocytosis Signaling	0.000617
**4**	**Antigen Presentation Pathway**	**0.001096**
5	p38 MAPK Signaling	0.001148
**6**	**Communication between Innate and Adaptive Immune Cells**	**0.003631**
7	Death Receptor Signaling	0.003631
**8**	**TREM1 Signaling**	**0.003631**
9	Oncostatin M Signaling	0.003631
10	Integrin Signaling	0.006761
11	Cdc42 Signaling	0.007079
**12**	**Type I Diabetes Mellitus Signaling**	**0.00912**
13	NF-κB Signaling	0.01
**14**	**Graft-versus-Host Disease Signaling**	**0.016218**
**15**	**IL-6 Signaling**	**0.017378**
**16**	**Role of Osteoblasts, Osteoclasts and Chondrocytes in Rheumatoid Arthritis**	**0.020893**
**17**	**Role of Macrophages, Fibroblasts and Endothelial Cells in Rheumatoid Arthritis**	**0.033884**
18	Cholecystokinin/Gastrin-mediated Signaling	0.043652
19	Agrin Interactions at Neuromuscular Junction	0.044668
**20**	**Altered T Cell and B Cell Signaling in Rheumatoid Arthritis**	**0.048978**
21	Virus Entry via Endocytic Pathways	0.048978
22	Actin Nucleation by ARP-WASP Complex	0.048978
**23**	**Dendritic Cell Maturation**	**0.048978**

Table S7: Canonical Pathways Associated with Mesenchymal Signature Genes and their Relation to those Associated with Immune Activation and IL13Rα2 Expression (FDR < 0.05). Immune-related pathways are shown in **bold red**, pathways common to IL13Rα2 expression in both patient cohorts and IL13Rα2-positive cell lines are highlighted, and additional pathways common to only IL13Rα2-positive cell lines are italicized.

	Ingenuity Canonical Pathways	FDR
**1**	**Hepatic Fibrosis / Hepatic Stellate Cell Activation**	**0.000005**
**2**	**TREM1 Signaling**	**0.000072**
**3**	**Dendritic Cell Maturation**	**0.000079**
4	***IL-6 Signaling***	***0.000132***
**5**	**HMGB1 Signaling**	**0.000132**
**6**	**IL-10 Signaling**	**0.000132**
7	Coagulation System	0.000155
8	*NF-κB Signaling*	*0.000269*
**9**	**Acute Phase Response Signaling**	**0.000331**
**10**	**Leukocyte Extravasation Signaling**	**0.000851**
11	N-Glycan Degradation	0.000851
12	Production of Nitric Oxide and Reactive Oxygen Species in Macrophages	0.000851
13	NF-κB Activation by Viruses	0.000955
14	LXR/RXR Activation	0.001230
**15**	**IL-15 Signaling**	**0.002455**
16	Integrin Signaling	0.002570
17	Agrin Interactions at Neuromuscular Junction	0.003311
**18**	**IL-4 Signaling**	**0.004266**
**19**	**Toll-like Receptor Signaling**	**0.004786**
20	PTEN Signaling	0.005495
21	VDR/RXR Activation	0.005623
**22**	**Natural Killer Cell Signaling**	**0.006607**
**23**	**B Cell Receptor Signaling**	**0.006607**
24	Glioma Invasiveness Signaling	0.008318
25	*Oncostatin M Signaling*	*0.008511*
**26**	**LPS/IL-1 Mediated Inhibition of RXR Function**	**0.010471**
27	Inhibition of Angiogenesis by TSP1	0.010715
28	JAK/Stat Signaling	0.012023
29	Ephrin Receptor Signaling	0.013804
30	Actin Cytoskeleton Signaling	0.013804
31	Atherosclerosis Signaling	0.014454
**32**	**T Helper Cell Differentiation**	**0.014454**
33	*p38 MAPK Signaling*	*0.015849*
**34**	**IL-3 Signaling**	**0.015849**
35	***Role of Osteoblasts, Osteoclasts and Chondrocytes in Rheumatoid Arthritis***	***0.015849***

36	Rac Signaling	0.015849
**37**	**Systemic Lupus Erythematosus Signaling**	**0.015849**
**38**	**IL-17 Signaling**	**0.015849**
**39**	**FLT3 Signaling in Hematopoietic Progenitor Cells**	**0.015849**
40	Endothelin-1 Signaling	0.015849
**41**	**Type I Diabetes Mellitus Signaling**	**0.015849**
42	Caveolar-mediated Endocytosis Signaling	0.015849
43	TGF-β Signaling	0.015849
44	TNFR1 Signaling	0.015849
45	MSP-RON Signaling Pathway	0.015849
46	Regulation of eIF4 and p70S6K Signaling	0.015849
47	Sphingosine-1-phosphate Signaling	0.016596
48	Role of Pattern Recognition Receptors in Recognition of Bacteria and Viruses	0.017378
**49**	**IL-12 Signaling and Production in Macrophages**	**0.019055**
**50**	**CD28 Signaling in T Helper Cells**	**0.023988**
51	Death Receptor Signaling	0.030200
52	Induction of Apoptosis by HIV1	0.031623
53	Intrinsic Prothrombin Activation Pathway	0.031623
**54**	**CTLA4 Signaling in Cytotoxic T Lymphocytes**	**0.031623**
55	PPAR Signaling	0.031623
56	Neuregulin Signaling	0.031623
57	*Virus Entry via Endocytic Pathways*	*0.031623*
58	Myc Mediated Apoptosis Signaling	0.033113
59	Axonal Guidance Signaling	0.033113
60	Type II Diabetes Mellitus Signaling	0.033113
**61**	**Role of NFAT in Regulation of the Immune Response**	**0.033113**
**62**	**PI3K Signaling in B Lymphocytes**	**0.033113**
63	MIF-mediated Glucocorticoid Regulation	0.035481
**64**	**T Cell Receptor Signaling**	**0.038019**
65	Hepatic Cholestasis	0.039811
66	Phospholipase C Signaling	0.039811
67	Macropinocytosis Signaling	0.039811
**68**	**Role of Macrophages, Fibroblasts and Endothelial Cells in Rheumatoid Arthritis**	**0.041687**
69	Colorectal Cancer Metastasis Signaling	0.044668
70	Erythropoietin Signaling	0.047863
**71**	**LPS-stimulated MAPK Signaling**	**0.047863**

Table S8: Canonical Pathways associated with Classical Signature Genes in Relation to those associated with Immune Activation and IL13Rα2 Expression (FDR < 0.05). The immune-related pathway is shown in **bold red**, and the pathway common to IL13Rα2 expression in patient cohorts and IL13Rα2-positive cell lines is highlighted.

	Ingenuity Canonical Pathways	FDR
1	Wnt/β-catenin Signaling	0.001122
2	Human Embryonic Stem Cell Pluripotency	0.003715
3	Corticotropin Releasing Hormone Signaling	0.005495
4	Colorectal Cancer Metastasis Signaling	0.005495
5	CREB Signaling in Neurons	0.010715
6	Glioma Signaling	0.010715
7	nNOS Signaling in Skeletal Muscle Cells	0.010715
8	Axonal Guidance Signaling	0.011220
**9**	**Role of Macrophages, Fibroblasts and Endothelial Cells in Rheumatoid Arthritis**	**0.013490**
10	Glioblastoma Multiforme Signaling	0.015136
11	Molecular Mechanisms of Cancer	0.032359
12	Factors Promoting Cardiogenesis in Vertebrates	0.032359
13	Ovarian Cancer Signaling	0.033113
14	Glutamate Receptor Signaling	0.037154

Table S9: Canonical Associated with Proneural Signature Genes and their Relation to those Associated with Immune Activation and IL13Rα2 Expression (FDR < 0.05). No immune-related pathways or pathways common to IL13Rα2 expression in patient cohorts and IL13Rα2-positive cell lines were reported.

	Ingenuity Canonical Pathways	FDR
1	Reelin Signaling in Neurons	0.008710
2	Cyclins and Cell Cycle Regulation	0.008710
3	Cell Cycle: G1/S Checkpoint Regulation	0.010471
4	Wnt/β-catenin Signaling	0.035481
5	Amyloid Processing	0.035481
6	Molecular Mechanisms of Cancer	0.035481
7	Semaphorin Signaling in Neurons	0.035481

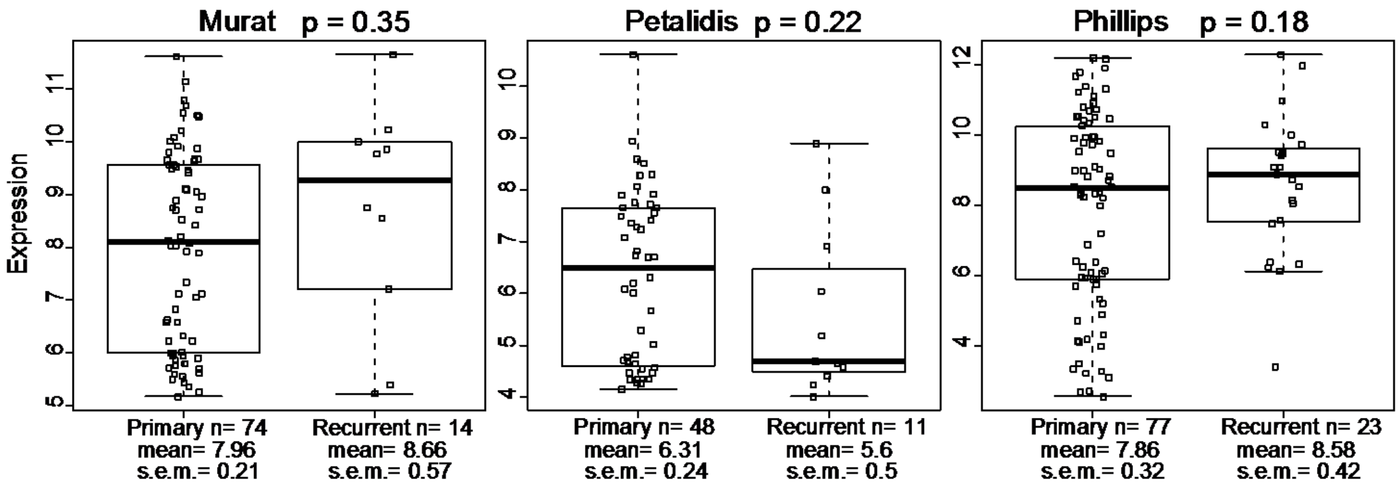

Figure S1. Expression of IL13Rα2 does not change with tumor recurrence.
Shown are box plots (median bar, 25th and 75th percentile box and error bars representing 10th and 90th percentiles) of IL13Rα2 expression in primary (left) and recurrent (right) tumors. Differences in expression levels were not statistically significant ($p > 0.05$; t-test).

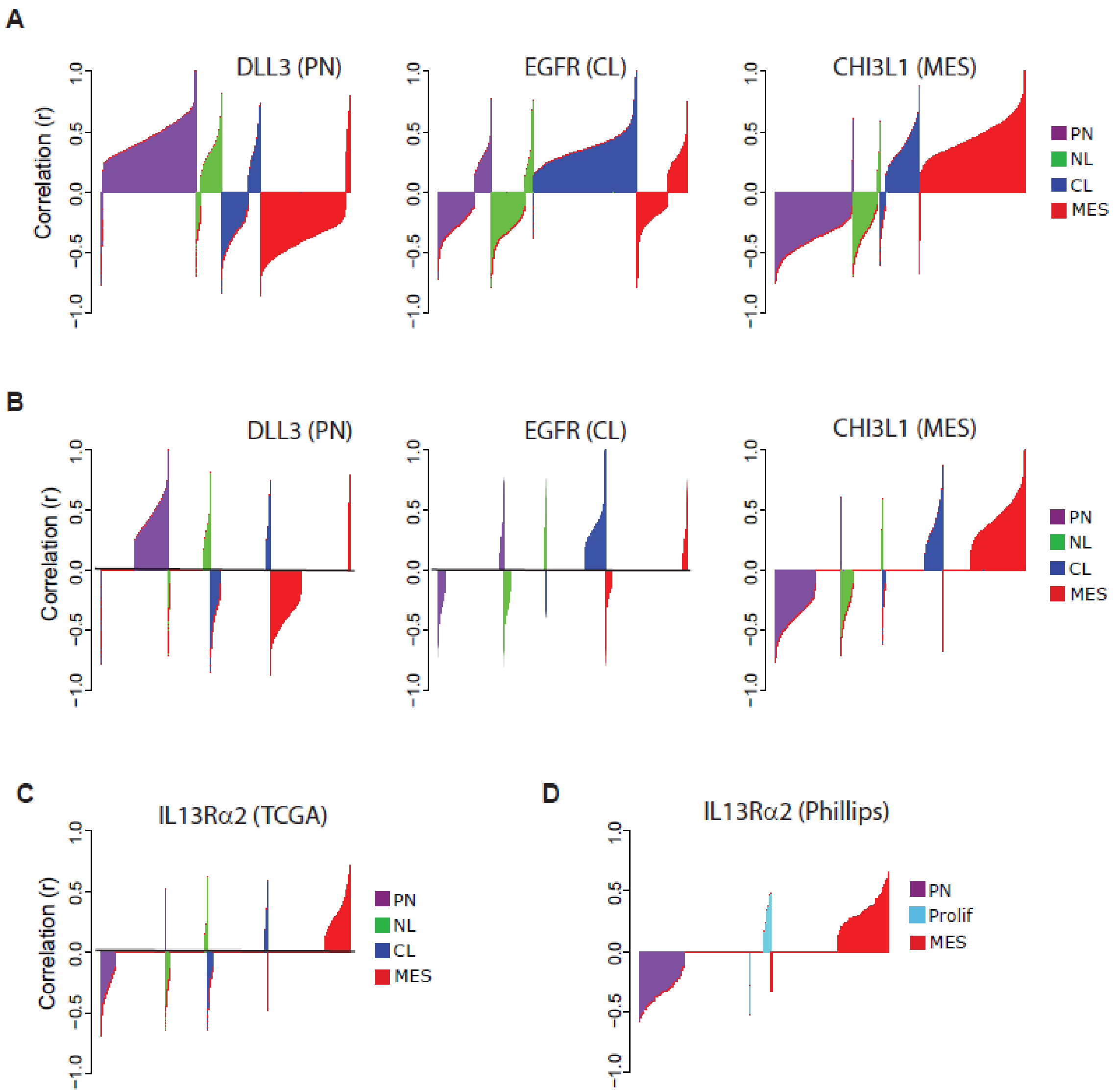

Figure S2. Validation of Silhouette plot analysis correlating expression of an individual gene with glioma subtypes. (A) Silhouette plots of Pearson correlation coefficients (r) for all significant correlations (FDR < 0.05) between individual signature genes (DLL3, EGFR and CHI3L1) representative of the proneural (PN), classical (CL), and mesenchymal (MES) subtypes, respectively. Correlations are sorted based upon GBM subtype and then ordered by increasing correlation coefficient values. **(B)** Silhouette plots as depicted in **(A)**, except non-significant correlations are also plotted (spaces). **(C, D)** Silhouette plots of Pearson correlation coefficients (r) for all significant (FDR < 0.05) and non-significant correlations between IL13Rα2 and probes for genes defining glioma subtypes as defined by **(C)** Verhaak et al., and **(D)** Phillips et al.

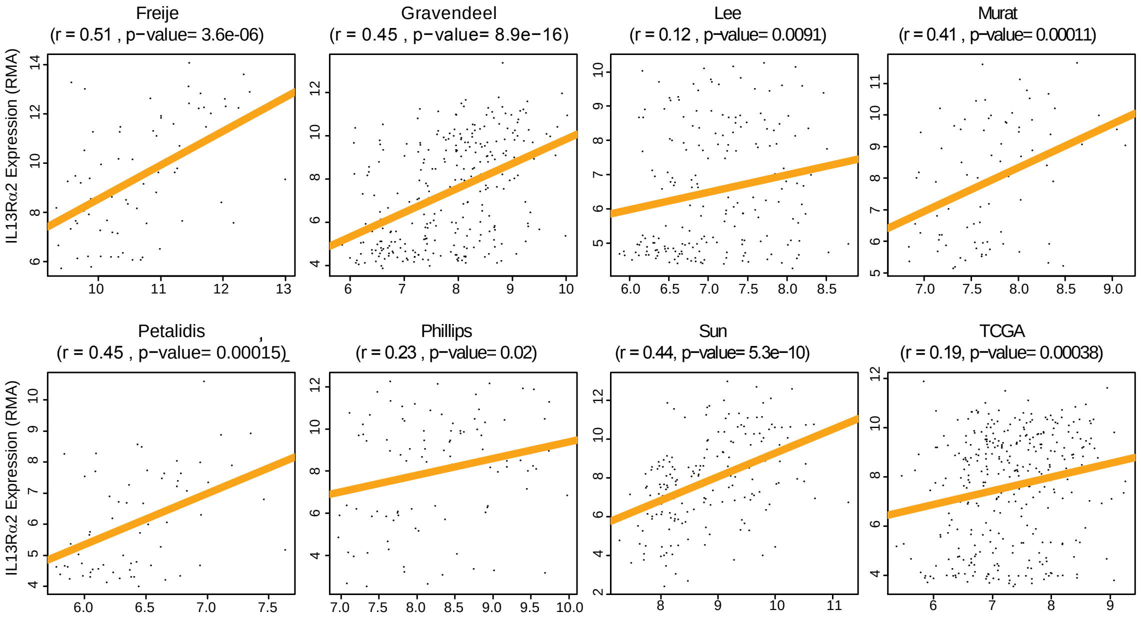

Figure S3. Correlation of IL13Rα2 versus average mesenchymal signature gene expression for individual study cohorts. Shown are scatter plots of IL13Rα2 expression in individual tumors (vertical axis) plotted against average mesenchymal signature gene expression defined using the 173 mesenchymal gene probes of Verhaak for each of the 8 glioma cohorts. The orange line indicates the results of fitting a linear regression model to these data; $p < 0.05$ for all cohorts.

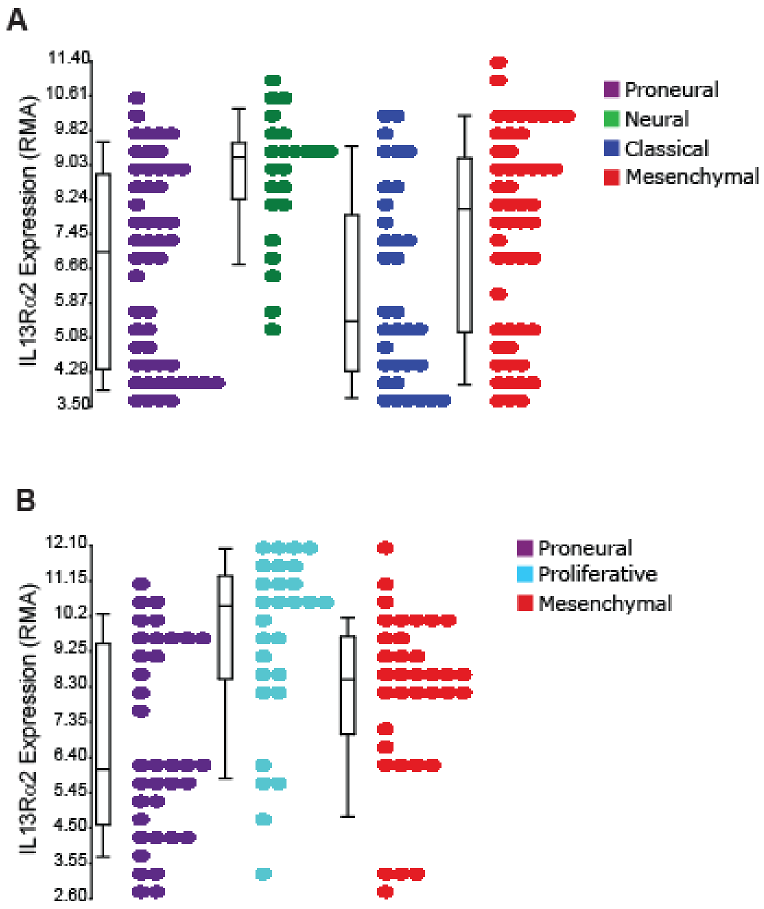

Figure S4. Dot plots showing distributions of RMA-normalized IL13Rα2 expression levels across molecularly-defined subtypes.
IL13Rα2 expression levels were evaluated with respect to molecularly-defined subtypes for (**A**) Verhaak et al. [1, 9], and (**B**) Phillips et al. [2]. The Verhaak dataset was obtained from TCGA (HT HG-U133A arrays) with subtype annotations for the 176 sample training set. The Phillips dataset was obtained from GEO (GSE4271) and subtype annotations from Phillips et al. [2]. Dot-plots of RMA-normalized IL13Rα2 expression levels (Partek® Genomics Suite™) showed a 1.68-fold increase (p=0.00015) in expression between mesenchymal and proneural tumors for Verhaak subtypes, and a 2.44-fold increase (p=0.028) in mesenchymal versus proneural expression for the Philips subtypes. Fold-change values were calculated based on the least-squares mean and p-values calculated based on 1-way ANOVA with appropriate linear contrast (Partek® Genomics Suite™).
While IL13Rα2 expression is significantly enriched in mesenchymal subclass tumors as defined by both studies, its expression is not limited to mesenchymal tumors. IL13Rα2 is also highly expressed in the proliferative subclass of Phillips et al., consistent with results of several approaches including the Silhouette analysis (Figure 2D), and the correlation plots (Figure 2F), suggesting that proliferative tumors are the next closest subtype associating with IL13Rα2. The high expression of IL13Rα2 in the neural subclass of Verhaak et al. likely reflects a bias in this small training dataset, because it is not found for the average neural expression in the larger dataset (Figure 2E), and no consistent association was suggested by our silhouette or PCA analyses (Figure 2A and 2C).

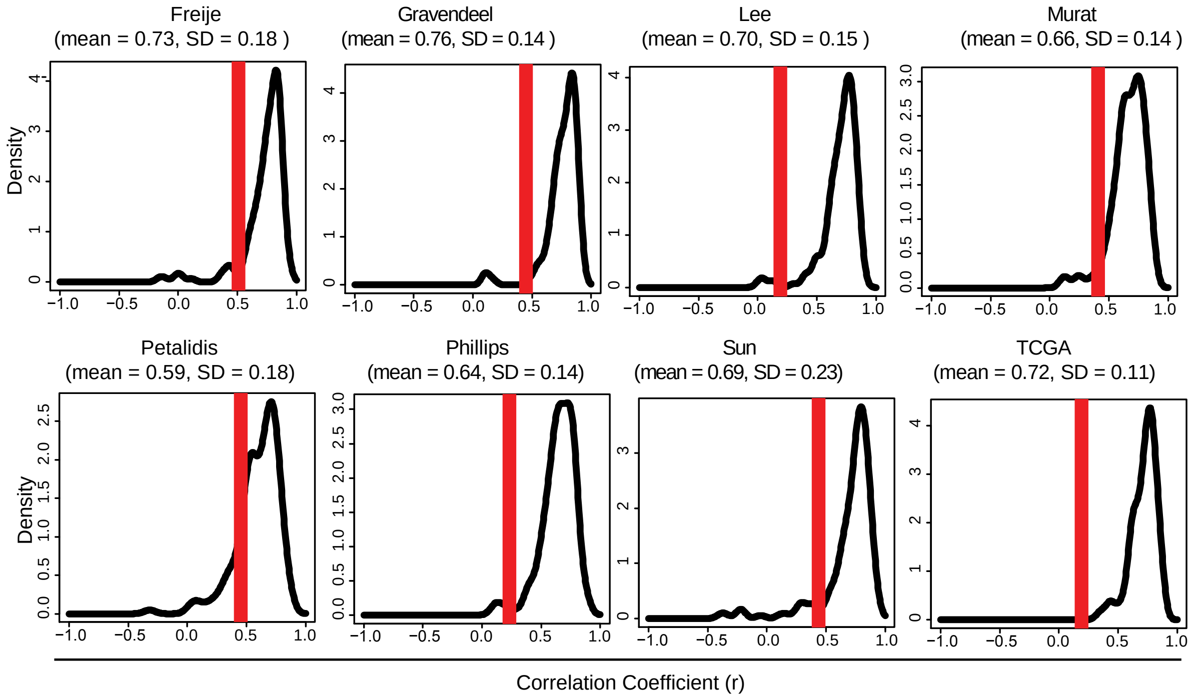

Figure S5. Density of correlation coefficients for average mesenchymal signature gene expression per study.

Shown are correlation coefficients for IL13Rα2 (red) versus average mesenchymal signature gene expression (black histogram) for eight study cohorts [2-9] (Table 1). The plots illustrate that the correlation of IL13Rα2 expression compared to known mesenchymal signature genes is close to but typically1-2 standard deviations below the average mesenchymal signature gene. IL13Rα2 correlation for each study is shown in red, and for comparison mean and standard deviations for the mesenchymal signature gene correlations are shown above each plot.

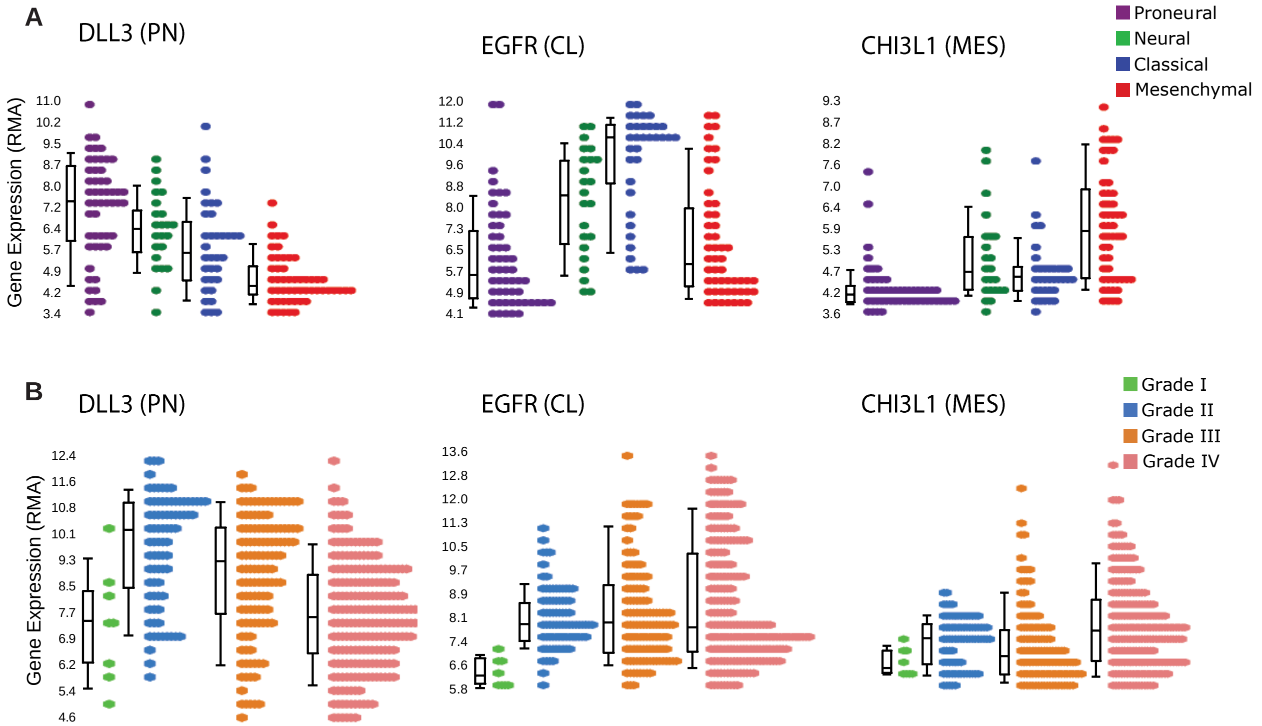

Figure S6. Comparison of a DLL3, EGFR, and CHI3L1 gene expression with respect to glioma subclass and malignancy grade.

(**A**) Dot plots for RMA-normalized expression of DLL3, EGFR and CHI3L1 representative of the proneural (PN), classical (CL), and mesenchymal (MES) molecular subtypes, respectively. Dot plots were produced using Partek Genomics Suite using expression data from Verhaak et al. [1]. DLL3 expression is represented by probe 219537_x_at (1/1 DLL3 probes), EGFR expression is represented by probe 201984_s_at (1/6 EGFR probes), and CHI3L1 expression is represented by probe 216546_s_at (1/3 CHI3L1 probes). Mesenchymal versus proneural fold-change values: DLL = -5.9-fold change ($p = 8.8 \times 10^{-16}$), EGFR = 6.6-fold change ($p = 7.6 \times 10^{-14}$), CHI3L1 = 3.2-fold change ($p = 2.3 \times 10^{-12}$). (**B**) RMA-normalized signature gene expression across WHO Grades using a dataset combining those of Sun et al. [4] and Gravendeel et al. [3]. Grade IV versus grade I/II/III fold-change values: DLL = -2.0-fold change ($p = 2.3 \times 10^{-5}$), EGFR = 2.1-fold change ($p = 2.4 \times 10^{-5}$), CHI3L1 = 1.6-fold change ($p = 6.5 \times 10^{-8}$). Grade III/IV versus grade I/II fold-change values: DLL = -1.2-fold change ($p = 0.40$), EGFR = 2.4-fold change ($p = 1.2 \times 10^{-4}$), CHI3L1 = 1.4-fold change ($p = 0.0061$).

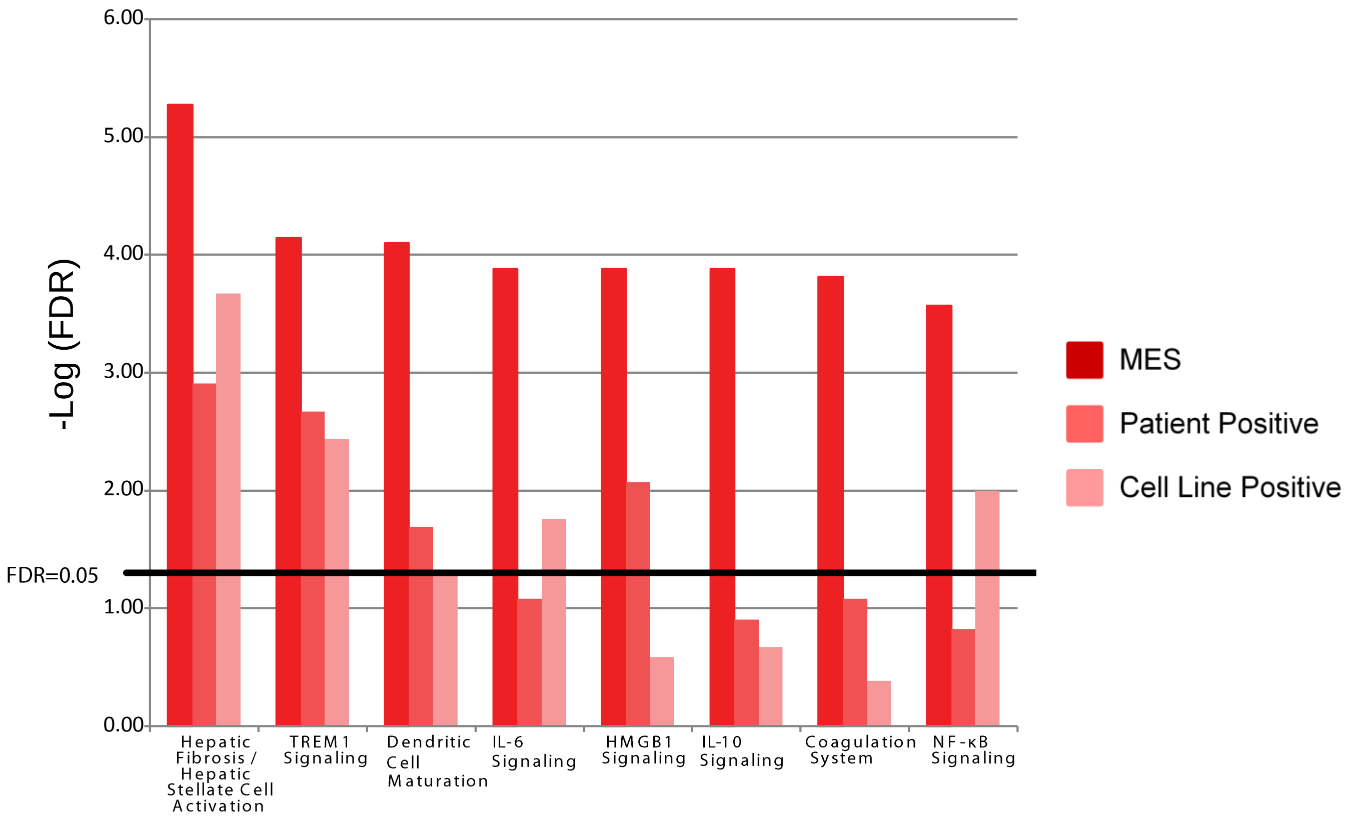

Figure S7. Pathways identified by IPA for mesenchymal signature genes are also highly enriched for genes correlated with IL13Rα2 over-expression.
Pathways with the largest IPA enrichments (shown as –log(FDR) values on the vertical axis) for mesenchymal signature genes, compared to IPA enrichment of these same pathways for genes positive correlated with IL13Rα2 expression in patient cohorts, and genes up-regulated in IL13Rα2-positive cell lines. The horizontal black line marks the threshold for pathways enriched with an FDR < 0.05. Hepatic Fibrosis / Hepatic Stellate Cell Activation and TREM1 Signaling pathways are significantly enriched in IPA analyses of mesenchymal signature genes, up-regulated patient sample genes, and genes up-regulated in IL13Rα2-positive cell lines (Table 2 and 3). In addition, IL-6, HMGB1 and NF-κB signaling pathways are significantly enriched in two of these three groups.

A

B

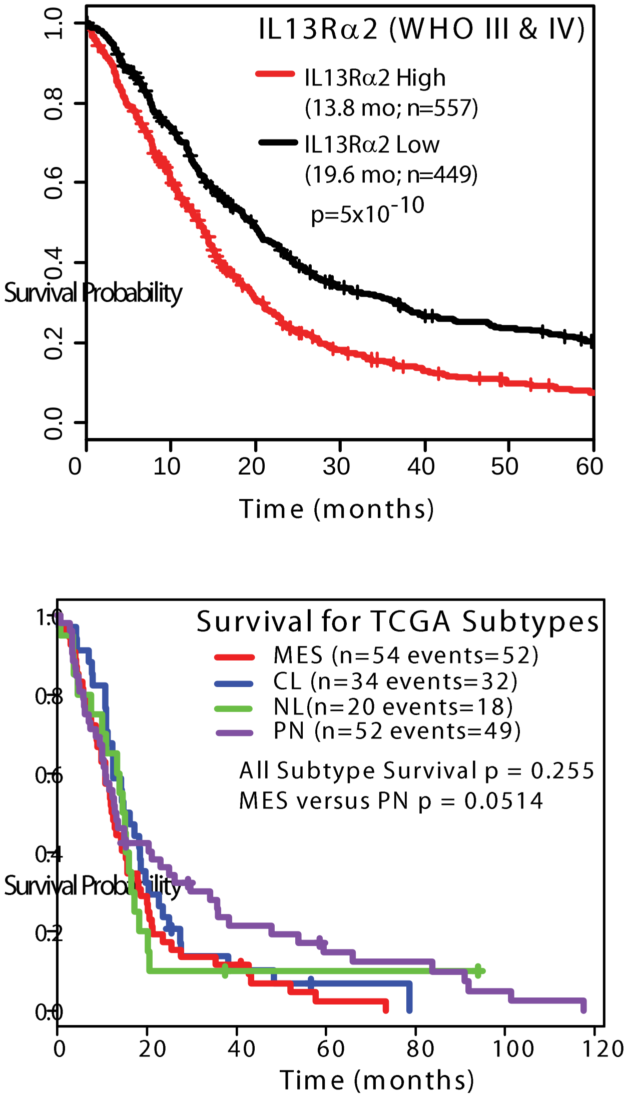

Figure S8: Glioma survival analyses.

(**A**) Kaplan-Meier survival plot comparing high-grade (WHO III and IV) patient tumors with ‘high’ and ‘low’ IL13Rα2 expression among 8 patient cohorts evaluated in Figure 4 [2-9]. IL13Rα2 expression levels are associated with a 5.8 month difference in median survival time ($p = 5.0x10^{-10}$; log-rank test) with a 13.8 month median survival time for ‘high’ IL13Rα2 expression (CI 95%: 12.4-14.8 months, n = 557) as compared to 19.6 months for ‘low’ IL13Rα2 expression (CI 95%: 16.9 – 22.2 months, n = 443).

(**B**) Kaplan-Meier survival plot for patients identified as having mesenchymal (MES), classical (CL), neural (NL), or proneural (PN) tumors as defined by Verhaak et al. [1] TCGA training dataset. No significant difference in the Kaplan-Meier curves ($p > 0.05$) is seen when all 4 subtypes are examined, but p approaches 0.05 when only mesenchymal and proneural subtypes are compared.

References for Supplemental Tables and Figures:

1. Verhaak RGW, Hoadley KA, Purdom E, Wang V, Qi Y, Wilkerson MD, et al. Integrated Genomic Analysis Identifies Clinically Relevant Subtypes of Glioblastoma Characterized by Abnormalities in PDGFRA, IDH1, EGFR, and NF1. Cancer cell 2010;17:98-110.
2. Phillips HS, Kharbanda S, Chen R, Forrest WF, Soriano RH, Wu TD, et al. Molecular subclasses of high-grade glioma predict prognosis, delineate a pattern of disease progression, and resemble stages in neurogenesis. Cancer cell 2006;9:157 - 73.
3. Gravendeel LAM, Kouwenhoven MCM, Gevaert O, de Rooi JJ, Stubbs AP, Duijm JE, et al. Intrinsic Gene Expression Profiles of Gliomas Are a Better Predictor of Survival than Histology. Cancer Research 2009;69:9065-72.
4. Sun L, Hui A-M, Su Q, Vortmeyer A, Kotliarov Y, Pastorino S, et al. Neuronal and glioma-derived stem cell factor induces angiogenesis within the brain. Cancer cell 2006;9:287-300.
5. Lee Y, Scheck A, Cloughesy T, Lai A, Dong J, Farooqi H, et al. Gene expression analysis of glioblastomas identifies the major molecular basis for the prognostic benefit of younger age. BMC Medical Genomics 2008;1:52.
6. Freije WA, Castro-Vargas FE, Fang Z, Horvath S, Cloughesy T, Liau LM, et al. Gene expression profiling of gliomas strongly predicts survival. Cancer Res 2004;64:6503 - 10.
7. Murat A, Migliavacca E, Gorlia T, Lambiv WL, Shay T, Hamou M-F, et al. Stem Cell-Related "Self-Renewal" Signature and High Epidermal Growth Factor Receptor Expression Associated With Resistance to Concomitant Chemoradiotherapy in Glioblastoma. Journal of Clinical Oncology 2008;26:3015-24.
8. Petalidis LP, Oulas A, Backlund M, Wayland MT, Liu L, Plant K, et al. Improved grading and survival prediction of human astrocytic brain tumors by artificial neural network analysis of gene expression microarray data. Molecular Cancer Therapeutics 2008;7:1013-24.
9. The Cancer Genome Atlas Research Network. Comprehensive genomic characterization defines human glioblastoma genes and core pathways. Nature 2008;455:1061-8.
